# Plant-Microbe Interaction: Mining the Impact of Native Bacillus amyloliquefaciens WS-10 on Tobacco Bacterial Wilt Disease and Rhizosphere Microbial Communities

**DOI:** 10.1128/spectrum.01471-22

**Published:** 2022-08-01

**Authors:** Waqar Ahmed, Zhenlin Dai, Jinhao Zhang, Shichen Li, Ayesha Ahmed, Shahzad Munir, Qi Liu, Yujiao Tan, Guanghai Ji, Zhengxiong Zhao

**Affiliations:** a College of Resources and Environment, Yunnan Agricultural University, Kunming, Yunnan, China; b State Key Laboratory for Conservation and Utilization of Bio-Resources in Yunnan, Yunnan Agricultural University, Kunming, Yunnan, China; c Key Laboratory of Agro-Biodiversity and Pest Management of Ministry of Education, Yunnan Agricultural University, Kunming, Yunnan, China; d College of Agronomy and Biotechnology, Yunnan Agricultural University, Kunming, Yunnan, China; e College of Tobacco Science, Yunnan Agricultural University, Kunming, Yunnan, China; USDA - San Joaquin Valley Agricultural Sciences Center

**Keywords:** biological control, disease incidence, *Ralstonia solanacearum*, microbiome, plant pathogens, plant-microbe interaction

## Abstract

Ralstonia solanacearum, the causative agent of bacterial wilt disease, has been a major threat to tobacco production globally. Several control methods have failed. Thus, it is imperative to find effective management for this disease. The biocontrol agent Bacillus amyloliquefaciens WS-10 displayed a significant control effect due to biofilm formation, and secretion of hydrolytic enzymes and exopolysaccharides. In addition, strain WS-10 can produce antimicrobial compounds, which was confirmed by the presence of genes encoding antimicrobial lipopeptides (*fengycin*, *iturin*, *surfactin*, and *bacillomycinD*) and polyketides (*difficidin*, *bacilysin*, *bacillibactin*, and *bacillaene*). Strain WS-10 successfully colonized tobacco plant roots and rhizosphere soil and suppressed the incidence of bacterial wilt disease up to 72.02% by reducing the R. solanacearum population dynamic in rhizosphere soil. Plant-microbe interaction was considered a key driver of disease outcome. To further explore the impact of strain WS-10 on rhizosphere microbial communities, V3-V4 and ITS1 variable regions of 16S and ITS rRNA were amplified, respectively. Results revealed that strain WS-10 influences the rhizosphere microbial communities and dramatically changed the diversity and composition of rhizosphere microbial communities. Interestingly, the relative abundance of genus *Ralstonia* significantly decreased when treated with strain WS-10. A complex microbial co-occurrence network was present in a diseased state, and the introduction of strain WS-10 significantly changed the structure of rhizosphere microbiota. This study suggests that strain WS-10 can be used as a novel biocontrol agent to attain sustainability in disease management due to its intense antibacterial activity, efficient colonization in the host plant, and ability to transform the microbial community structure toward a healthy state.

**IMPORTANCE** The plant rhizosphere acts as the first line of defense against the invasion of pathogens. The perturbation in the rhizosphere microbiome is directly related to plant health and disease development. The introduction of beneficial microorganisms in the soil shifted the rhizosphere microbiome, induced resistance in plants, and suppressed the incidence of soilborne disease. *Bacillus* sp. is widely used as a biocontrol agent against soilborne diseases due to its ability to produce broad-spectrum antimicrobial compounds and colonization with the host plant. In our study, we found that the application of native Bacillus amyloliquefaciens WS-10 significantly suppressed the incidence of tobacco bacterial wilt disease by shifting the rhizosphere microbiome and reducing the interaction between rhizosphere microorganisms and bacterial wilt pathogen.

## INTRODUCTION

Nicotiana tabacum Linne, commonly known as tobacco, is an important cash crop globally and in China (1). China is the world's largest producer of tobacco leaves, with about 1.08 million ha area reserved for tobacco cultivation, and produces approximately 2.1 million tons of tobacco leaf annually (2). Yunnan Province is in southwest China and is famous for its exclusive climatic conditions, suitable for producing high-yield and better-quality tobacco (3). Yunnan contributes about 50% of China's total tobacco leaf yield, with an annual production of approximately 750,000 tons (4). However, flue-cured tobacco production has been influenced by several insect pests and disease-causing agents.

Bacterial wilt disease caused by a soilborne pathogenic bacterium, Ralstonia solanacearum, poses a major constrain on tobacco production worldwide (5). R. solanacearum is the second most destructive phytopathogenic bacteria, infecting 254 plant species belonging to 54 botanical families (6, 7). It survives in the soil and on infected plant parts as a saprophyte for up to 40 years and spreads from plant to plant through irrigation water (8). It enters the plant through the spots of primary and secondary roots development, and wounds formed on the roots due to mechanical operations (9). Infected plants commonly show yellowing and wilting of leaves, root rot, and discoloration of parenchyma cells, resulting in dark black necrotic spots on the stem (10).

Tobacco bacterial wilt disease is prevalent in 14 out of 22 tobacco-growing areas of China, with an average disease incidence of about 15 to 35%. However, it can reach up to 75% in certain conditions when it occurs as a disease complex with the Phytophthora nicotianae (11). Yield losses can reach 100% in areas of continuous mono-cropping and high humidity (8). Several measures in the form of cultural control, application of pesticides, crop rotation, and soil disinfection have been executed to manage bacterial wilt disease (12), but none of these strategies has produced satisfactory results (13). The excessive use of pesticides is hazardous to the environment, develops resistance to the pathogen, and poses public health concerns (14).

Soil health and rhizospheric microbial diversity play an imperative role in the occurrence of soilborne diseases (1, 5). Soil amendments with calcium, molybdenum (15), and biochar (1) alleviate the incidence of bacterial wilt disease by reducing the pathogen load and improving the soil physicochemical properties and rhizosphere microbial diversity. In recent years, biological control through biocontrol agents (BCAs), i.e., Acinetobacter sp., *Bacillus* sp., Enterobacter sp., *Lysobacter* sp., and Pseudomonas sp., has been considered an efficient and eco-friendly approach to mitigate soilborne diseases. These BCAs suppress soilborne disease through versatile mechanisms of niche exclusion, direct antagonism, colonization, and the production of several secondary metabolites (13, 16).

Members of *Bacillus* sp. are well studied and widely used as BCAs against soilborne diseases. They produce broad-spectrum antimicrobial compounds, enhance plant growth, induce host resistance, and successfully colonize the host rhizosphere (17, 18). It has been reported that the combined application of B. amyloliquefaciens QL-5 (19), B. amyloliquefaciens SQR-7, and B. methylotrophicus SQR-29 (20) and bio-organic fertilizers significantly reduced the incidence of tomato and tobacco bacterial wilt disease. B. licheniformis Bl17 enhanced potato plant growth and suppressed bacterial wilt incidence by 41.31% (21). Similarly, B. amyloliquefaciens BZ6-1 was also found to reduce bacterial wilt incidence in peanuts by producing *fengycin* A and *surfactin* (22). In another study, B. velezensis FJAT-46737 isolated from the mountain soil significantly suppressed the incidence of tomato bacterial wilt by up to 96.2% and could secrete lipopeptides as well (23).

Our previous study demonstrated that B. amyloliquefaciens WS-10 isolated from the healthy tobacco plant rhizosphere soil enhanced the dry matter contents and suppressed bacterial wilt incidence by enriching functional diversity of rhizosphere microorganisms (24). However, gaps are still present related to the assembly and composition of the recruited rhizosphere microbiome against the pathogen in the presence of B. amyloliquefaciens WS-10. Thus, the current work could provide the early warning system to study the impact of B. amyloliquefaciens WS-10 on the assembly of the rhizosphere microbiome and the interaction between BCAs, pathogen, and rhizosphere microbes to unravel the underlying biocontrol mechanisms against tobacco bacterial wilt disease. In addition, this study also provides the fundamentals of plant-microbial crosstalk for host-pathogen interaction and microbe-microbe interaction to mitigate bacterial wilt incidence on flue-cured tobacco.

## RESULTS

### Assessment of biofilm formation, hydrolytic enzymes, and exopolysaccharide production ability.

The activities of amylase, cellulase, protease synthesis, exopolysaccharide (EPS) production, and biofilm-forming ability of biocontrol strain B. amyloliquefaciens WS-10 were assessed after 72 h of incubation (Fig. S1 in Supplemental File 1). The results demonstrated that strain WS-10 could form a biofilm, secrete amylase and cellulase, and produce EPS (2.255 mg/mL), whereas protease activity was not detected in the assay (Fig. S2A).

### Analysis of antimicrobial biosynthesis genes.

Antimicrobial compounds, including lipopeptides and polyketides, are known to be directly involved in antibiosis. The presence of genes involved in the antimicrobial biosynthesis pathway is an indication of the antimicrobial potential of a bacterium. We detected the presence of lipopeptides and polyketides biosynthesis genes in the genome of strain WS-10 (Fig. S2). It was confirmed that the genome of strain WS-10 contains all four lipopeptides (*fenA*, *ituC*, *srfA*, and *bmyA*) and four polyketides (*dfnA*, *bacA*, *dhbA*, and *beaS*) genes (Fig. S2).

### Assessment of disease index and population dynamic of Ralstonia solanacearum WS-001.

**FIG 1:**
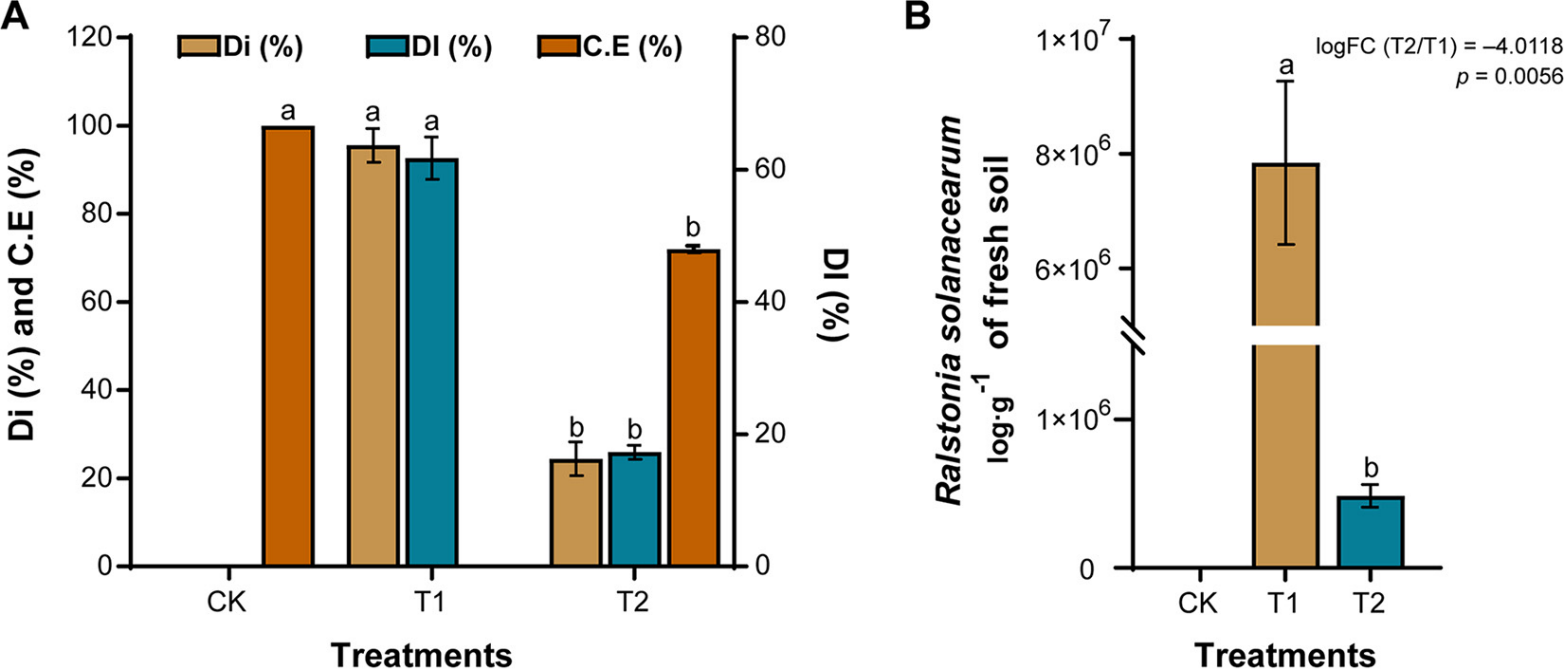
Analyses of disease index and population dynamics of Ralstonia solanacearum WS-001 in rhizosphere soil. Disease index (A) and R. solanacearum WS-001 population log/g of fresh soil (B). Disease index (DI), disease incidence (Di), control effect (CE), application of water (CK), application of R. solanacearum WS-001 (T1), and combined application of R. solanacearum WS-001 and Bacillus amyloliquefaciens WS-10 (T2). Different lowercase letters on error bars show significant differences among treatments according to Duncan's multiple range test at *P* < 0.05.

Disease index (DI), disease incidence (Di), and R. solanacearum WS-001 gene copy number/g in soil were assessed at the end of the experiment under different treatments (Fig. 1). It was observed that the application of strain WS-10 significantly suppressed the incidence of bacterial wilt disease (based on disease index) with a biocontrol effect of about 72.02% (Fig. 1A) and reduced the R. solanacearum WS-001 population in the rhizosphere of the tobacco plant (log fold change [FC] = −4.0118, *P* = 0.0056) (Fig. 1B).

### Colonization ability of Bacillus amyloliquefaciens WS-10 in tobacco roots and rhizosphere soil.

**FIG 2:**
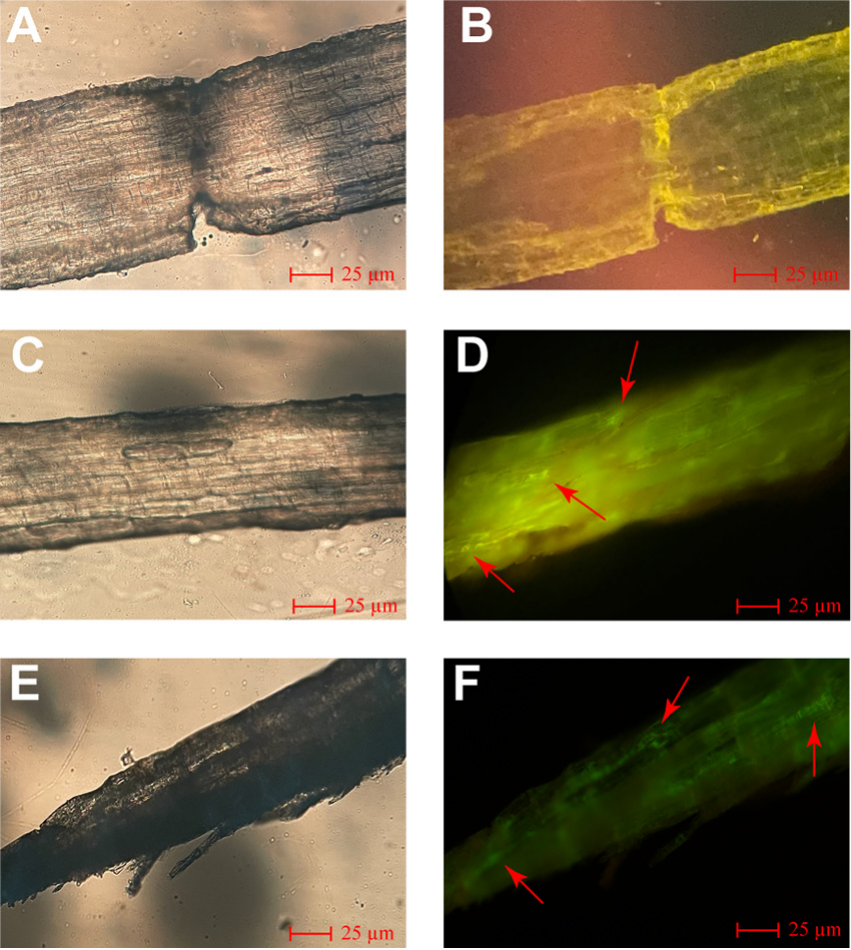
Fluorescence micrographs of flue-cured tobacco roots colonized by *gfp*-labeled Bacillus amyloliquefaciens WS-10. Fluorescence micrographs of control after 7 days under dark (A) and bright (B) fields. Fluorescence micrographs of treatment after 7 days under dark (C) and bright (D) fields. Fluorescence micrographs of treatment after 21 days under dark (E) and bright (F) fields. Here: Control; B. amyloliquefaciens WS-10 without *gfp* and treatment; *gfp*-labeled B. amyloliquefaciens WS-10. Colonization of *gfp*-labeled B. amyloliquefaciens WS-10 cells is shown by red arrows.

Colonization of *gfp*-labeled WS-10 was assessed 7 and 21 days postinoculation under a confocal laser scanning microscope and after 21 days in the rhizosphere soil via plate culture method. Seven days postinoculation, the *gfp*-labeled WS-10 was visualized mainly on the surface of tobacco roots. After 21 days, the *gfp*-labeled WS-10 was evenly distributed in intracellular tissues (Fig. 2). A serial dilution (10^−2^) of rhizosphere soil inoculated with *gfp*-labeled WS-10 was prepared. Around 200 μL solution was spread on LB medium plates containing kanamycin (50 μg/mL), and plates were incubated at 28°C for 48 h. The bacteria were visible due to *gfp* fluorescence under UV light (Fig. S3). Whereas no fluorescence was observed in the roots and rhizosphere soil of the control group. These results indicate that *gfp*-labeled WS-10 successfully colonized the rhizosphere soil and transferred it into roots from the soil through irrigation water.

### Effect of Ralstonia solanacearum WS-001 and Bacillus amyloliquefaciens WS-10 on flue-cured tobacco microbiome assembly.

A total of 9 rhizosphere soil samples were subjected to an Illumina MiSeq platform for 16S and ITS amplicon sequencing. The amplicon sequencing resulted in 720,192/661,375 raw reads with an average length of 416 bp/255 bp per sample through the amplification of 16S and ITS1 *rRNA*, respectively. After quality control and the chimeras filter, 700,828 bacterial and 653,474 fungal effective reads with an average of 77,869 bacterial and 70,608 fungal reads per sample were obtained. The effective reads were clustered at a 97% sequence similarity level for taxonomic annotation, and 9,332 bacterial and 3,049 fungal operational taxonomic units (OTUs) were recovered from all 9 samples with an average of 1037 bacterial and 339 fungal OTUs per sample (Table S3). Analysis of raw reads (number), effective reads (number), and OTUs (number) revealed that R. solanacearum WS-001 and strain WS-10 had no significant impact on bacterial microbiome assembly. However, the microbiome assembly of fungi significantly changed under different treatments.

### Analyses of rarefaction curve and operational taxonomy units.

Rarefaction curves and unique OTUs generated from all 9 samples under different treatments through amplification of V3-V4/ITS1 variable regions of bacterial/fungal rRNA are shown in Fig. S4. Rarefaction curves generated to access the richness of bacterial (Fig. S4A) and fungal (Fig. S4B) communities under different treatments showed that a reasonable number of reads were obtained in all samples for bacterial and fungal communities. Analysis of OTUs revealed that a total of 1188 bacterial (Fig. S4C) and 875 fungal (Fig. S4D) specific OTUs were recovered from the rhizosphere soil samples of different treatments. The number of unique bacterial OTUs retrieved was almost similar among the treatments (CK, T1, and T2). Whereas the number of unique fungal OTUs in treatment T2 (160 OTUs) was significantly higher than T1 (74 OTUs) and CK (83 OTUs).

### Assessment of microbial community diversity and structure.

**FIG 3:**
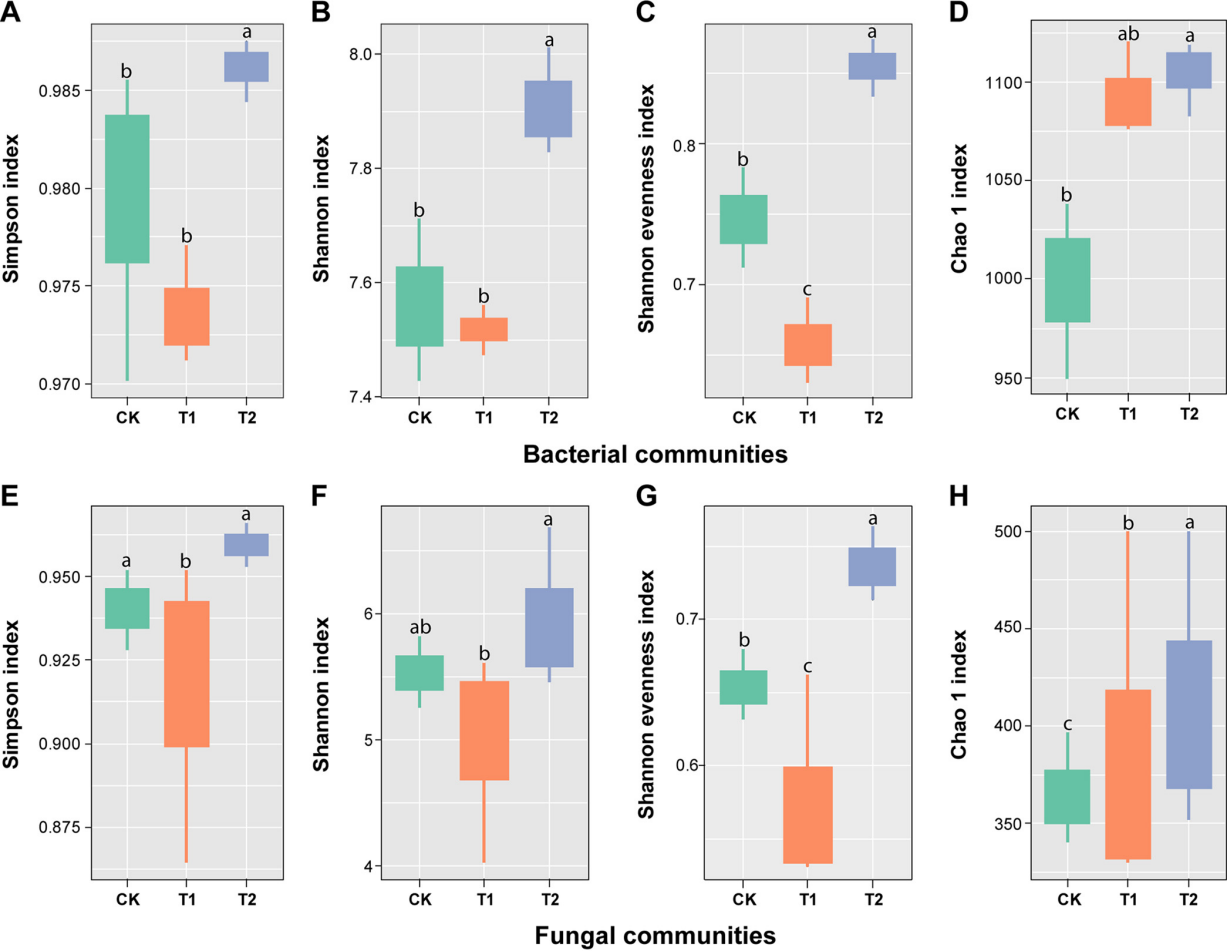
Box plot showing the alpha diversity indices of bacterial (top) and fungal (bottom) communities under different treatments. (A to D) Alpha diversity indices of bacterial communities Simpson, Shannon, Shannon evenness, and Chao 1, respectively. (E to H) Alpha diversity indices of fungal communities Simpson, Shannon, Shannon evenness, and Chao 1, respectively. Application of water (CK), application of Ralstonia solanacearum WS-001 (T1), and combined application of R. solanacearum WS-001 and Bacillus amyloliquefaciens WS-10 (T2). Different lowercase letters on each box plot represent the significant differences among treatments according to the Wilcoxon test at *P* < 0.05.

We assessed the within-sample diversity (alpha diversity indices; Simpson, Shannon, Shannon evenness, and Chao 1) of bacterial and fungal communities at the 97% threshold level (Fig. 3 and Table S4) under different treatments (CK, T1, and T2). Simpson and Shannon indexes for bacterial communities in treatment T2 were found significantly higher than CK and T1 (T2 > CK and T1, Wilcoxon test, *P* < 0.05), but no significant difference was observed between CK and T1 (Wilcoxon test, *P* > 0.05) (Fig. 3A and B). A significant difference was observed for the Shannon evenness index of bacterial communities among all treatments, and the values of the Shannon evenness index decreased in the order T2 > CK > T1 (Wilcoxon test, *P* < 0.05) (Fig. 3C). Chao 1 index for bacterial communities under treatment T2 was significantly higher than CK (T2 > CK, Wilcoxon test, *P* < 0.05); whereas no significant difference was observed between CK and T1 (Wilcoxon test, *P* > 0.05), and T1 and T2 (Wilcoxon test, *P* > 0.05) (Fig. 3D). For fungal communities, the Simpson index of T2 and CK was significantly higher than T1 (T2 and CK > T1; Wilcoxon test, *P* < 0.05). In contrast, no significant difference was observed between T2 and CK (Wilcoxon test, *P* > 0.05) (Fig. 3E). Shannon index of T2 for fungal communities was found significantly higher than T1 (T2 > T1, Wilcoxon test, *P* < 0.05), but no significant difference was observed between CK and T1 (Wilcoxon test, *P* > 0.05), and CK and T2 (Wilcoxon test, *P* > 0.05) (Fig. 3F). A significant difference was observed for Shannon evenness (T2 > CK > T1) and Chao 1 (T2 > T1 > CK) indexes of fungal communities among all treatments (Wilcoxon test, *P* < 0.05) (Fig. 3G and H).

**FIG 4:**
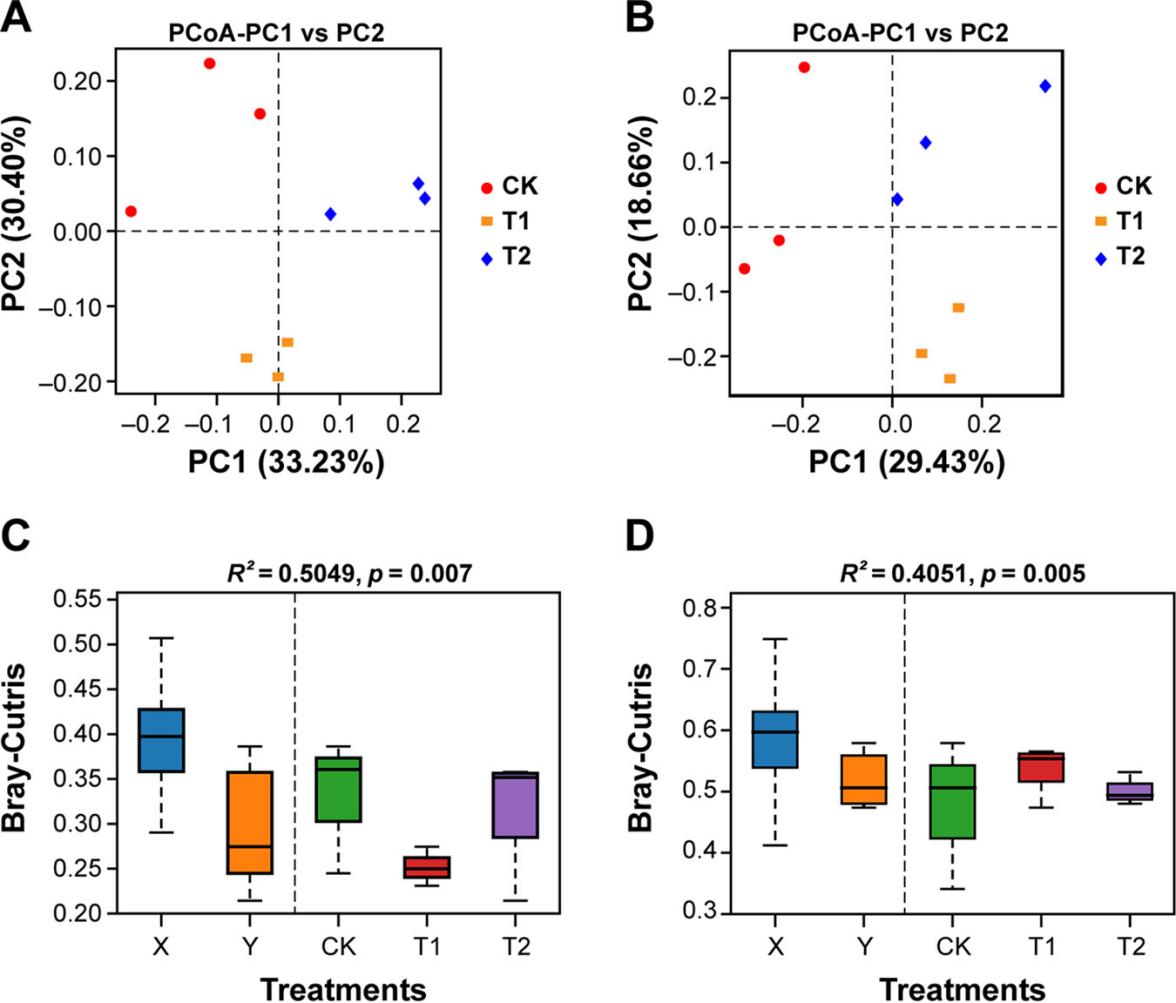
Principal coordinate analysis (PCoA) and permutational multivariate analysis of variance (PERMANOVA) based on the Bray-Curtis distance matrix demonstrating the separation between soil bacterial and fungal communities under different treatments. PCoA for bacterial (A) and fungal (B) communities. Overall differences among bacterial (C) and fungal (D) communities are shown by a pairwise PERMANOVA (*P* < 0.01). Application of water (CK), application of Ralstonia solanacearum WS-001 (T1), and combined application of R. solanacearum WS-001 and Bacillus amyloliquefaciens WS-10 (T2). Overall differences in microbial community composition between the treatments (X) and within the treatments (Y).

Principal coordinate analysis (PCoA) based on the Bray-Curtis dissimilarity matrix was used to assess the changes in the structure of bacterial and fungal communities under different treatments (Fig. 4). A clear separation could be observed between bacterial (Fig. 4A) and fungal (Fig. 4B) communities under different treatments (CK, T1, and T2). According to the PCoA results, the first two axes explained 33.23 and 30.40% of the total variation for bacterial communities (Fig. 4A), and 29.43 and 18.66% of the total variation for fungal communities (Fig. 4B). Permutational multivariate analysis of variance (PERMANOVA) of pairwise distances between bacterial (*R*^2^ = 0.5049, *P* < 0.01; Fig. 4C) and fungal (*R*^2^ = 0.4051, *P* < 0.01; Fig. 4D) communities indicated that the rhizosphere microbiome differed significantly under different treatments (Table S5). Bray-Curtis dissimilarity matrix heatmaps for bacterial and fungal communities structure under different treatments are shown in Fig. S5. This suggested that the application of strain WS-10 significantly influences the diversity and structure of bacterial and fungal communities and changed the rhizosphere microbiome toward a healthier state in the presence of R. solanacearum WS-001.

### Relative abundance analysis of microbial community composition at phyla level.

**FIG 5:**
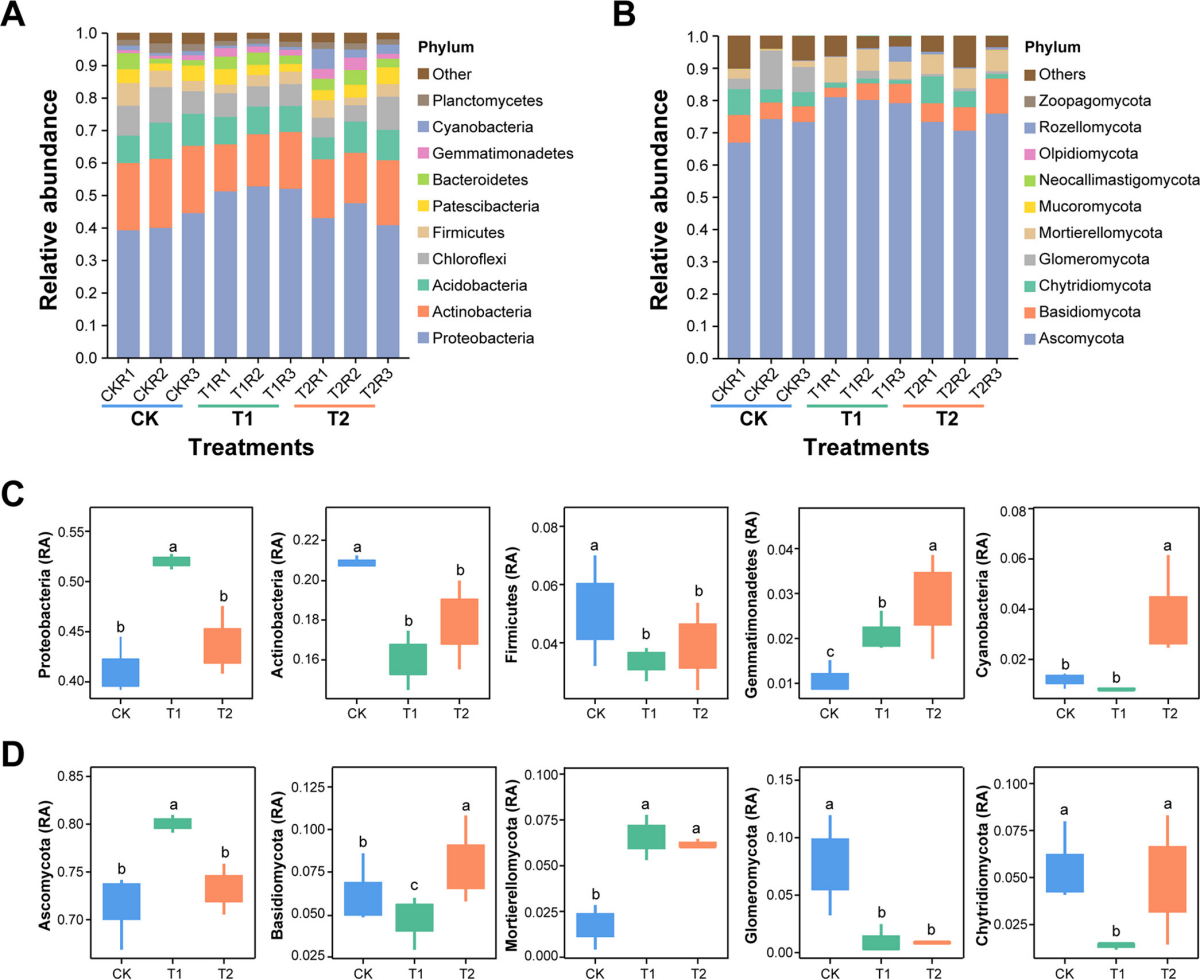
The dynamics of most dominant bacterial and fungal communities at the phyla level. Relative abundance bar plots for the top 10 most abundant bacterial (A) and fungal (B) phyla. The box plot shows the significant difference and relative abundance of the differentially abundant bacterial (C) and fungal (D) phyla under different treatments. The lowercase letters on each box plot display significant differences among treatments (Wilcoxon test, *P* < 0.05). Application of water (CK), application of Ralstonia solanacearum WS-001 (T1), and combined application of R. solanacearum WS-001 and Bacillus amyloliquefaciens WS-10 (T2).

The relative abundance (RA) of the top 10 bacterial and fungal phyla under different treatments (CK, T1, and T2) is shown in Fig. 5 and Table S6. At the phyla level, 97.35 and 94.04% of the total OTUs were classified in to top 10 bacterial and fungal phyla, respectively, in terms of RA (Fig. 5A and B). Bacterial phyla such as Proteobacteria, Actinobacteria, Acidobacteria, Chloroflexi, and Firmicutes dominated the soil bacterial communities and accounted for around 84.55% of the total soil bacteriome (Fig. 5A and Table S6). Soil fungal communities were dominated by phyla such as Ascomycota, Basidiomycota, and Mortierellomycota and accounted for around 86.01% of total soil fungal communities (Fig. 5B and Table S6). Proteobacteria was found in high RA in the rhizosphere soil of T1 compared with CK and T2 (Wilcoxon test, *P* < 0.05; Fig. 5C). There were several phyla such as Actinobacteria, Acidobacteria, Chloroflexi, Firmicutes, and Planctomycetes were present in high RA and unique to rhizosphere soil of CK than T1 and T2 (Wilcoxon test, *P* < 0.05; Fig. 5C and Table S6). In contrast, the RA of Gemmatimonadetes and Cyanobacteria was significantly higher in T2 compared with CK and T1 (Wilcoxon test, *P* < 0.05; Fig. 5C and Table S6). Fungal phyla such as Ascomycota and Basidiomycota had significantly higher RAs and were present as unique phyla in the rhizosphere soil of T1 and T2 compared with CK or T2 and CK or T1, respectively (Wilcoxon test, *P* < 0.05; Fig. 5D and Table S6). Mortierellomycota had significantly higher RA in T1 and T2 compared with CK (Wilcoxon test, *P* < 0.05; Fig. 5D and Table S6). In contrast, the RA of Glomeromycota were significantly increased in CK than in T1 and T2, and Chytridiomycota was present in high RA in CK and T2 compared with T1 (Wilcoxon test, *P* < 0.05; Fig. 5D and Table S6).

### Relative abundance analysis of microbial community composition at the genera level.

**FIG 6:**
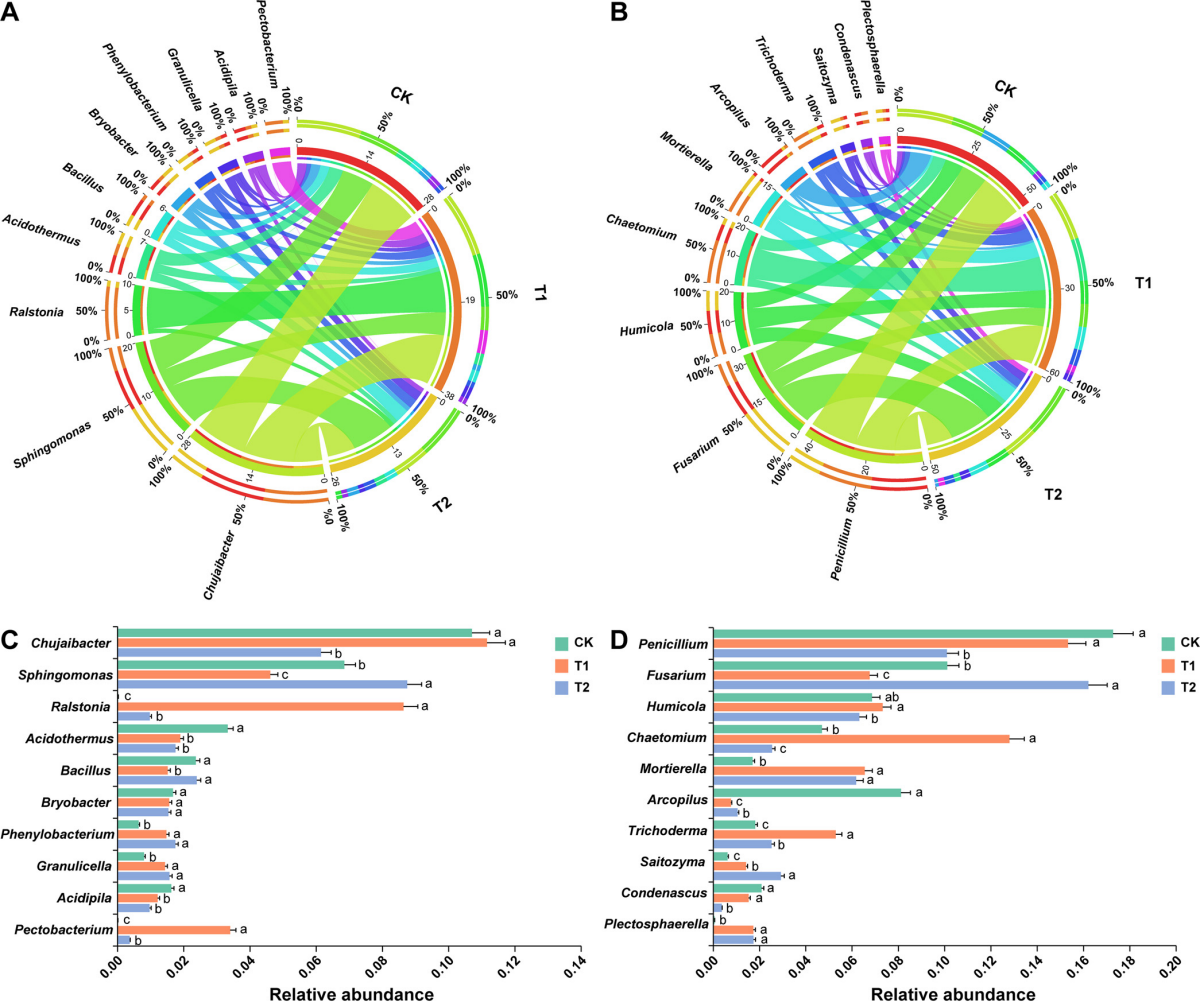
Changes in the relative abundance of bacterial and fungal communities at the genera level. Chord diagram showing the interrelationships between relative abundance of top 10 bacterial (A) and fungal (B) genera under different treatments. The change in the width of the color bands indicates the change in the relative abundance of bacterial and fungal communities. Bar plots show the significant differences in the relative abundance of most abundant bacterial (C) and fungal (D) genera under different treatments. Significant differences among treatments are shown by different lowercase small letters on the error bars (Wilcoxon test, *P* < 0.05). Application of water (CK), application of Ralstonia solanacearum WS-001 (T1), and combined application of R. solanacearum WS-001 and Bacillus amyloliquefaciens WS-10 (T2).

At the genera level, the patterns of taxonomic distribution and RA differences for bacteria and fungi under different treatments (CK, T1, and T2) became more evident (Fig. 6 and Table S7). A chord diagram was generated to show the interrelationship between the RA of the 10 most dominant bacterial (Fig. 6A) and fungal (Fig. 6B) communities at the genera level under different treatments. In contrast, the significant differences in the RA of most abundant bacterial (Fig. 6C) and fungal (Fig. 6D) communities at the genera level are shown by bar plots (Wilcoxon test, *P* < 0.05). *Chujaibacter* was the most dominant and significantly abundant in the rhizosphere soil of CK and T1 compared to T2, whereas *Sphingomonas* was present in high RA in T2 than CK and T1 (Wilcoxon test, *P* < 0.05; Fig. 6C). Surprisingly, the RA of *Ralstonia* and *Pectobacterium* significantly decreased in the order T1 > T2 > CK after the application of strain WS-10 (Wilcoxon test, *P* < 0.05; Fig. 6C). However, no significant difference was observed in the RA of *Bacillus* in CK and T2 (Wilcoxon test, *P* > 0.05) but was found to be significantly higher than T1 (Wilcoxon test, *P* < 0.05; Fig. 6C). *Penicillium* has a high RA and most abundant in the rhizosphere soil of CK and T1 than T2. In contrast, Fusarium was significantly dominant with T2 compared to CK and T1, and its RA decreased in the order T2 > CK > T1 (Wilcoxon test, *P* < 0.05; Fig. 6D). However, the genera *Chaetomium* and *Trichoderma* were more abundant in T1 than CK and T2 (Wilcoxon test, *P* < 0.05; Fig. 6D). This suggested that the bacterial and fungal community composition significantly changed under different treatments. The results of 16S high-throughput sequencing at the genera level for genus *Ralstonia* agreed with the qPCR results of gene copy number of R. solanacearum WS-001 that application of biocontrol strain WS-10 reduced the population of bacterial wilt pathogen R. solanacearum in the rhizosphere of tobacco plants.

### Characteristics of flue-cured tobacco microbiome co-occurrence network.

**FIG 7:**
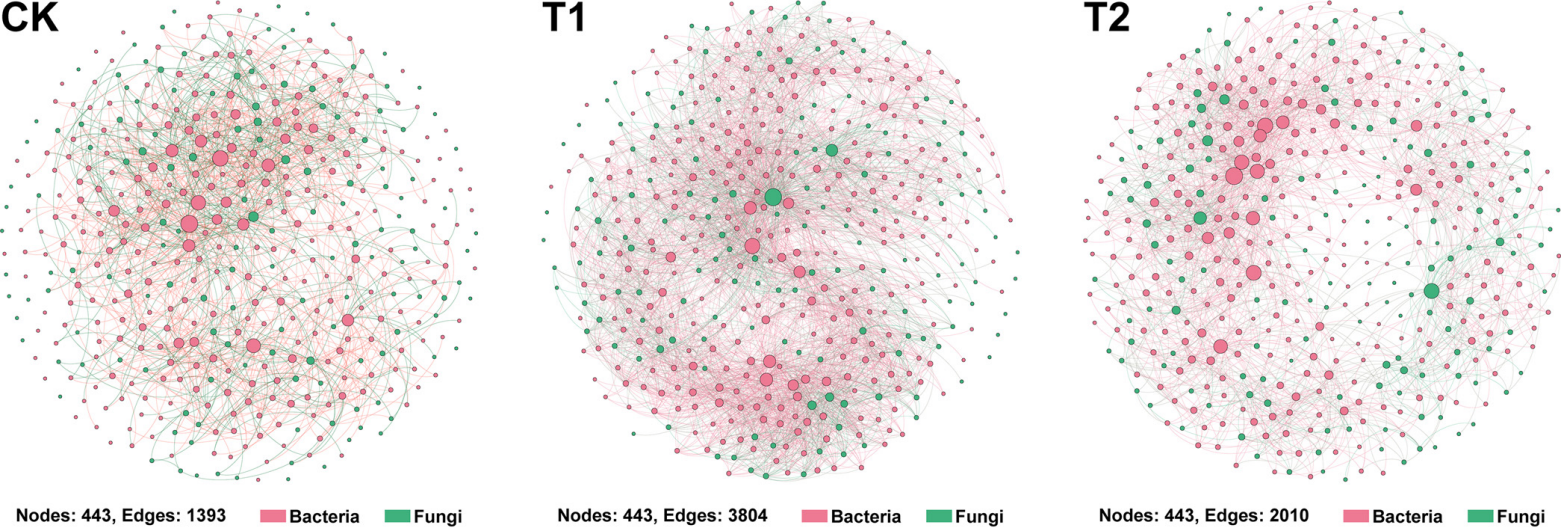
Co-occurrence networks analysis of bacterial and fungal communities at genus level under different treatments. Nodes represent microbial genera, and edges represent the interaction between microbes within a specific treatment. Application of water (CK), application of Ralstonia solanacearum WS-001 (T1), and combined application of R. solanacearum WS-001 and Bacillus amyloliquefaciens WS-10 (T2).

A microbial co-occurrence network was constructed for bacterial and fungal OTUs at the genus level for each treatment to investigate the effect of R. solanacearum WS-001 and strain WS-10 on the microbiome of flue-cured tobacco (Fig. 7). Nodes and edges represent the individual microbial genera and pairwise correlations between the microorganism (nodes), respectively, showing the biological or biochemical interactions between microbes within a network. The number of nodes was found to be the same (nodes = 443) under all treatments (CK, T1, and T2), whereas the number of edges was significantly higher in treatment T1 (edges = 3804) > T2 (edges = 2010) > CK (edges = 1393) (Fig. 7). Comparing the co-occurrence network among treatments (number of edges) showed that a more complex microbial interaction network existed in T1 compared with T2 and CK. This indicated that a facilitative interaction took place between R. solanacearum WS-001 and compatible microbes, which led to disease development. Whereas the application of biocontrol strain WS-10 weakened the interaction between pathogen and microbes, making the microbiome of flue-cured tobacco healthier closer to CK. Our results of the co-occurrence network were in accordance with the findings of qPCR and 16S microbial diversity analysis that the application of biocontrol strain WS-10 reduced the population of R. solanacearum WS-001 and improved the microbial community diversity and structure toward a healthier state.

### Correlation analysis.

**FIG 8:**
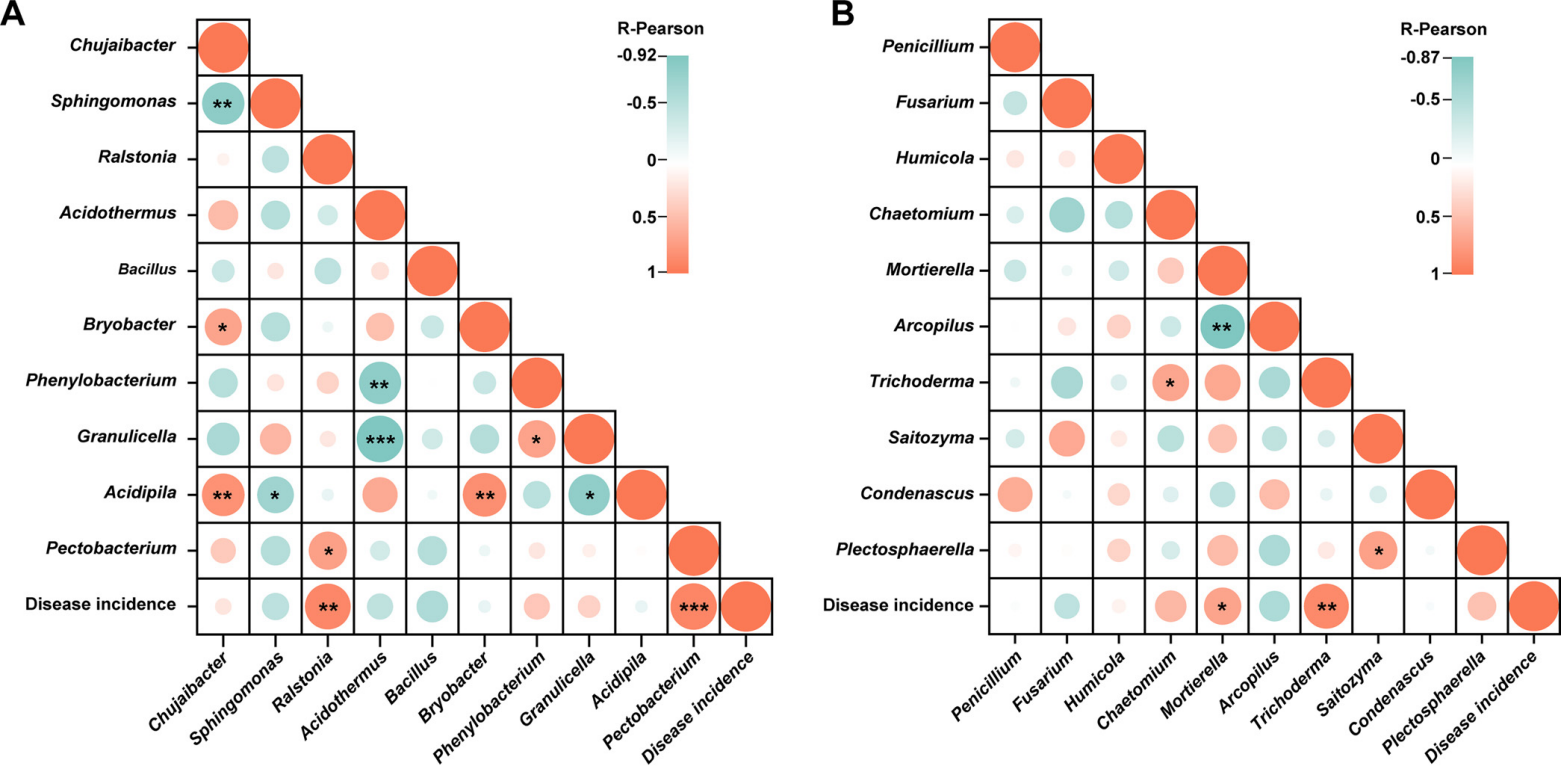
Correlation analysis between bacterial-fungal genera and disease incidence according to Pearson correlation coefficient (PCC, *P* < 0.05). PCC between bacterial genera and disease incidence (A), and PCC between fungal genera and disease incidence (B). Asterisks indicates significant differences (*, *P* < 0.05; **, *P* < 0.01; ***, *P* < 0.001).

Based on the results of the co-occurrence network, to further explore the influence of these microorganisms on disease incidence, a correlation analysis was performed at the genera level according to the Pearson correlation coefficient (PCC; Fig. 8). The PCC results revealed that bacterial genera *Ralstonia* and *Pectobacterium* (Fig. 8A) and fungal genera *Mortierella* and *Tichoderma* (Fig. 8B) were positively correlated (*P* < 0.05) with the disease incidence. This suggested that *Pectobacterium* may increase the population of *Ralstonia*, and *Mortierella* and *Tichoderma* may provide a conducive environment that accelerates the disease development. In contrast, the genus *Bacillus* was negatively correlated (*P* > 0.05) with disease incidence, which showed that *Bacillus* inhibits the growth of *Ralstonia*, thereby reducing the disease incidence.

## DISCUSSION

Ralstonia solanacearum has become a matter of great concern for the tobacco industry worldwide (1). Due to its wide geographical distribution, broad host range, high species complex, and persistent nature, it is extremely challenging to control this disease with absolute results (25). The application of pesticides causes environmental pollution and poses public health concerns. Thus, eco-friendly approaches are urgently required. Plants are habitats for diverse microbial communities which play a key role in health and safety. Therefore, biological control through host-specific biocontrol agents (BCAs) in the form of endophytes and rhizobacteria provides a great opportunity for sustainable agriculture (13).

Traditionally, a single BCA is used to mitigate the incidence of a specific plant pathogen, which is not sufficient to control the incidence of the disease complex (occurrence of one or two diseases together as black shank and bacterial wilt). However, screening of BCAs with broad-spectrum antimicrobial activity may target several pathogens of the same host plant (26). *In planta* assay, strain WS-10 could mitigate the incidence of bacterial wilt by up to 72.02% by reducing the population load of R. solanacearum WS-001 in the rhizosphere of the flue-cured tobacco plants. Pseudomonas aeruginosa NXHG29 had strong antagonistic activity against P. nicotianae and R. solanacearum *in vitro* and dramatically reduced the black shank and bacterial wilt incidence in a pot experiment (27), which is in congruence with our results.

Pathogens employ various strategies to infiltrate, escape, and thrive in host plants. However, extracellular hydrolytic enzymes secreted by the BCAs act as the first line of defense against pathogen invasion (28), promote plant growth, and decompose organic matter (29). It is reported that BCAs, including *Bacillus* sp. and Pseudomonas sp., can produce hydrolytic enzymes with strong antagonistic activity against several phytopathogens (30, 31). Similarly, it is reported that a biofilm-forming bacterium has good adaption to various environmental conditions and protects the host against pathogen infection (32). Exopolysaccharides (EPS) are termed “adhesive polymers,” which help bacteria to successfully colonize the host and are also considered the major factor influencing the biofilm-forming ability of microbes (33). Our results demonstrated that strain WS-10 can produce hydrolytic enzymes, EPS, and form biofilm, which highlights it as a remarkable biocontrol agent.

The presence of lipopeptides and polyketides biosynthesis genes is very important for better antagonizing action (23, 34). Antimicrobial lipopeptides and polyketides compounds produced by bacteria can inhibit many pathogenic bacteria and fungi. The most-reported antimicrobial lipopeptides and polyketides are *fengycin* (*fenA*), *iturin* (*ituC*), *surfactin* (*srfA*), *bacillomycinD* (*bmyA*) and *difficidin* (*dfnA*), and *bacilysin* (*bacA*), *bacillibactin* (*dhbA*), and *bacillaene* (*beaS*), respectively (35, 36). These genes are involved in the biosynthesis of antimicrobial lipopeptides and polyketides and are directly correlated with the biocontrol efficacy of BCAs against several phytopathogens (37). Our study revealed that the genome of WS-10 carries four lipopeptides (*fenA*, *ituC*, *srfA*, and *bmyA*) and four polyketides (*dfnA*, *bacA*, *dhbA*, and *beaS*) genes, which allow it to produce diverse antimicrobial compounds. *In planta* assay, WS-10 significantly suppressed bacterial wilt incidence and inhibited the growth of R. solanacearum WS-001 in the rhizosphere of the tobacco plant. Our findings are in accordance with previous studies that *Bacillus* sp. mitigates the incidence of bacterial wilt disease by producing lipopeptides and polyketides (23, 38, 39).

The plant rhizosphere serves as a hot spot habitat for various microorganisms, those are directly involved in disease development and plant growth (40). This study reveals that R. solanacearum WS-001 significantly influences the rhizosphere microbiome of flue-cured tobacco, resulting in bacterial wilt disease development. Whereas the application of biocontrol strain WS-10 inhibits the growth of R. solanacearum WS-001, changing the rhizosphere microbiome toward a healthier state, and reducing the bacterial wilt incidence. Many previous studies have reported that B. amyoliquefaciens ZM9 and Y4 suppressed bacterial wilt incidence on flue-cured tobacco by changing the diversity and composition of the rhizosphere microbiota (41, 42). Results of high-throughput sequencing revealed that the application of strain WS-10 has a significant impact on the microbial community diversity and composition of the flue-cured tobacco plant. We found that the alpha diversity indices (Simpson, Shannon, and Shannon evenness) for soil bacterial and fungal communities were significantly higher under T2. PCoA of soil bacterial and fungal communities showed a clear separation between the treatments (CK, T1, and T2), indicating that bacterial and fungal community composition significantly differed under the application of strain WS-10, which is similar to the results of previous reports (41–43). In this study, bacterial phyla, such as Proteobacteria, Actinobacteria, Acidobacteria, Chloroflexi, and Firmicutes, and fungal phyla, including Ascomycota, Basidiomycota, Mortierellomycota, and Chytridiomycota, were significantly more abundant in all rhizosphere soil samples. Our results roughly corresponded to the results of previous reports that agricultural soils mainly consist of bacterial phyla Proteobacteria, followed by Acidobacteria, Actinobacteria, Bacteroidetes, Planctomycetes, Chloroflexi, and Verrucomicrobia (44–46) and fungal phyla Ascomycota followed by Basidiomycota, and Mortierellomycota or Chytridiomycota (43, 46).

The RA of genus *Pectobacterium* was higher in T1 and significantly decreased in the order T1 > T2 > CK under the application of biocontrol strain WS-10, which showed that the strain WS-10 has an antagonistic effect on *Pectobacterium*. Genus *Pectobacterium* is a well-known plant pathogen that causes blackleg in tobacco and soft rot in Chinese cabbage, potato, and other crops (47). Many previous studies reported that the application of *Bacillus* sp. BS107 (48), B. amyloliquefaciens KC-1 (49), and B. amyloliquefaciens Ar10 (50) suppressed the disease development caused by P. carotovorum in tobacco (blackleg), Chinese cabbage (soft rot), and potato (soft rot) through a mechanism of induced systemic resistance, direct antagonism and colonization in the host plant, and by producing antimicrobial compound (glycolipid), respectively. It is reported that fungal species can provide a conducive environment for bacterial wilt infection (51), and the incidence of bacterial wilt can reach up to 75% when the disease occurs with the soilborne pathogen Phytophthora nicotianae (8). Further, correlation analysis revealed that bacterial and fungal genera, including *Pectobacterium*, *Mortierella*, and *Trichoderma* were positively correlated (*P* < 0.05) with disease incidence, may enhance the population of *Ralstonia*, and provide a conducive environment for bacterial wilt pathogen, which results in disease development. However, the interaction between *Ralstonia*, *Pectobacterium*, *Trichoderma*, and *Mortierella* related to disease acceleration is unclear and warrants further study. Analysis of the co-occurrence network showed the complexity of microbial associations in T1 under pathogen invasion which indicates increased cooperation within a network. Additionally, the presence of the highest number of edges (3804) in T1 than CK (1393) and T2 (2010) indicates that *Ralstonia* can recruit other microbes and pathogens in the rhizosphere of tobacco plants which play an important role in disease acceleration. In contrast, a simple co-occurrence network was observed in T2, demonstrating that strain WS-10 acts as an antagonist, reduced the interaction between *Ralstonia* and other microbes, improves diversity and composition of rhizosphere microbiota, and transformed the rhizosphere microbiome toward a healthier state. Previous studies reported that pathogen invasion significantly affects the rhizosphere microbiome assembly, and the application of BCAs reshapes the structure and composition of the rhizosphere microbiome (43, 52, 53).

The biocontrol mechanisms of bacteria mainly involve the induction of host resistance, antibiosis, and competition for niche and nutrients through colonization (54, 55). Therefore, the ability of biocontrol bacteria to colonize efficiently in the host plant and rhizosphere is considered a prerequisite factor for BCAs to be successfully applied in the field for effective disease management (56, 57). To further investigate the biocontrol mechanism of strain WS-10 against bacterial wilt disease, a *gfp-*labeled WS-10 strain was constructed, and its colonization ability was confirmed in the flue-cured tobacco roots and rhizosphere through a confocal microscope and plate culture methods, respectively. The *gfp*-labeled WS-10 strain successfully colonized the tobacco plant's roots and rhizosphere soil, and colonization patterns were observed the same as for other BCAs reported in previous studies (27, 58). The colonization first occurred in the specified regions of primary roots, then advanced toward the elongation zone of secondary roots, and finally spread along the stem at 21 days postinoculation. Based on the above discussion, it becomes clear that strain WS-10 can colonize the tobacco plant and positively affects the rhizosphere microbiome, which changes the diversity and composition of soil microbiota and could recruit important groups of microbes that could play an important role in disease suppression.

**FIG 9:**
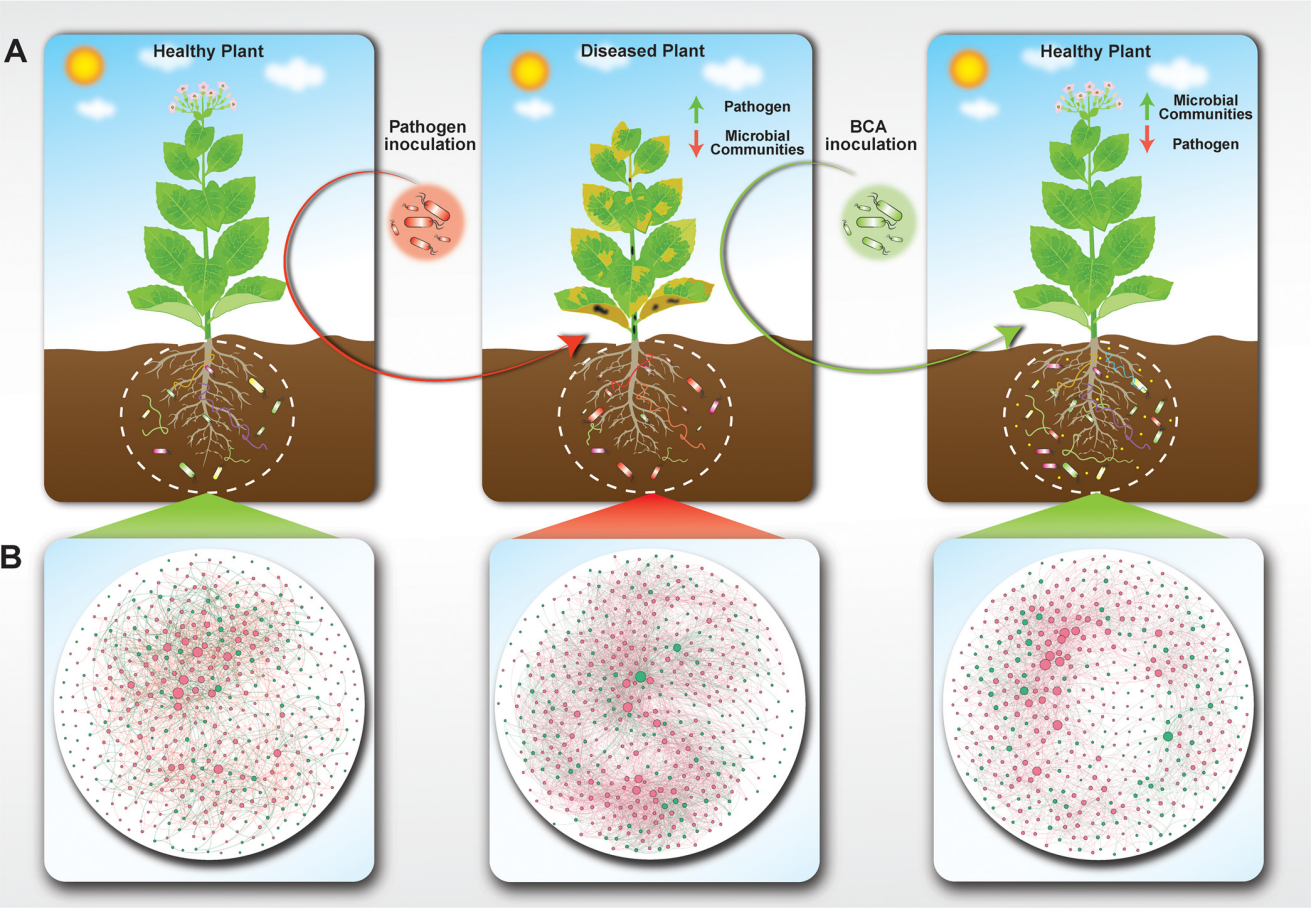
Plant-microbe interaction and biocontrol mechanism of biocontrol agents. (A) Above ground plant parts represent a healthy and diseased state of plants in the presence of biocontrol agents and pathogens. Healthy plants, plants under no pathogen invasion or treated with a biocontrol agent. Diseased plants; plants under pathogen invasion. (B) The rhizosphere of plants shows the complexity of the microbial co-occurrence network under a healthy and diseased state in the presence of biocontrol agents and pathogens. Biocontrol agents suppress disease incidence through mechanisms of antibiosis, colonization, and produce antimicrobial compounds, biofilm formation, and engineering of the rhizosphere microbiome. Positive interactions between plant-microbes and microbe-microbe induce resistance in plants and help plants in combating with pathogen. Negative interactions between plant-microbes and microbe-microbe result in the imbalance of the rhizosphere microbiome breaking the first line of defense against pathogen invasion, and enhancing pathogen population, and disease development.

In summary, we concluded that Bacillus amyloliquefaciens WS-10 reduced the Ralstonia solanacearum WS-001 population in the tobacco plant rhizosphere and suppressed the incidence of bacterial wilt disease by successful colonization and reshaping the rhizosphere microbiome (Fig. 9). B. amyloliquefaciens WS-10 modified the diversity and structure of the flue-cured tobacco rhizosphere microbiome. The complexity of the co-occurrence network showed that the microbial communities are more sensitive to R. solanacearum WS-001 and played a significant role in disease outcome. The suggested mechanisms of applied biocontrol agents were colonization, pathogen exclusion in the rhizosphere, hydrolytic enzyme production, biofilm formation, and reshaping of the rhizosphere microbiome. Genes related to antimicrobial lipopeptides, and polyketides were present in the genome of B. amyloliquefaciens WS-10, which showed that WS-10 can produce antimicrobial lipopeptides and polyketides. Therefore, B. amyloliquefaciens WS-10 is an ideal candidate to be developed as a biopesticide against bacterial wilt as well as other diseases of tobacco. Moreover, further studies on the extraction of these antimicrobial compounds and metabolomic analysis may provide new insights into the underlying biocontrol mechanisms of B. amyloliquefaciens WS-10.

## MATERIALS AND METHODS

### Bacterial strains and growth conditions.

Bacterial wilt pathogen Ralstonia solanacearum WS-001 (accession no. MW730714) and biocontrol strain Bacillus amyloliquefaciens WS-10 (accession no. MW730713) used in this study were already preserved in our laboratory “State Key Laboratory for Conservation and Utilization of Bio-resources in Yunnan,” Yunnan Agricultural University, China. R. solanacearum WS-001 was cultured on casamino acid-peptone-glucose (CPG) agar medium (casein hydrolysate 1 g/mL, peptone 10 g/mL, glucose 5 g/mL, agar 20 g/mL, and pH 7.0) (59), while strain WS-10 was grown on Luria-Bertani (LB) agar medium (tryptone 10 g/mL, yeast extract 5 g/mL, NaCl 10 g/mL, agar 18 g/mL, and pH 7.0) (60), and incubated at 28°C for 48 h. The pure culture of bacterial strains was stored at −80°C in a 50% glycerol solution (vol/vol) supplemented with 0.5% glucose.

### Analysis of different *in vitro* assays.

**(i) Assessment of hydrolytic enzyme production ability.** The amylase, cellulase, and protease production ability of strain WS-10 was observed on starch agar medium (SA), M9 minimal salt agar medium (M9), and skim milk powder (SMP) agar medium, respectively, as previously described by Agarwal et al. (38, 61). Briefly, 10 μL of strain WS-10 was cultured on SA, M9, and SMP medium and incubated at 28°C for 3 days. The SA and M9 plates were stained with Gram's iodine and 0.3% aqueous Congo red for 10 min, followed by washing with 75% ethanol to detect the amylase and cellulase activity, respectively. The presence of halo circles around bacterial colonies was taken as positive for amylase and cellulase activity, whereas the degradation of skim milk powder was recorded as positive for protease production.

**(ii) Exopolysaccharide production ability.** The exopolysaccharide (EPS) production ability of WS-10 was determined by the alcohol precipitation method, using the methodology of Liu et al. (62). Briefly, strain WS-10 was grown in nutrient broth (NB) medium (tryptone 5 g/ml, sucrose 10 g/ml, beef extract 3 g/ml, yeast extract 1 g/mL, and pH 7.0) at 28°C and 160 rpm for 3 days. The supernatant was collected through centrifugation at 12,000 rpm for 8 min, diluted with a 2-fold volume of 95% ethanol, and kept overnight at 4°C. The suspension was centrifuged at 12,000 rpm for 10 min. Crude EPS was collected as precipitates and weighed.

**(iii) Biofilm formation assay.** The crystal violet staining method was used to investigate the biofilm formation ability of strain WS-10 (61). Strain WS-10 was grown overnight in NB medium, ≈200 μL of bacterial suspension per well was added to 96-well plates and incubated at 28°C for 72 h, whereas sterilized distilled water (sdH_2_O) was used as control. After incubation, bacterial suspension was discarded from wells and washed thrice with 1 × PBS buffer solution. The wells were then stained with 0.5% crystal violet for 30 min, washed twice with sdH_2_O, and air-dried naturally. In the end, a 200 μL aliquot of 33% ice-cold acetic acid was added to each well and incubated at 28°C for 15 min. The light absorbance intensity of the solution was recorded at an optical density of 590 nm (OD_590_) with a microplate reader (Thermo Scientific Varioskan LUX).

### Analysis of antimicrobial lipopeptides and polyketides biosynthesis genes.

We confirmed the presence of four lipopeptides, *fengycin* (*fenA*), *iturin* (*ituC*), *surfactin* (*srfA*), and *bacillomycinD* (*bmyA*), and four polyketides, *difficidin* (*dfnA*), *bacilysin* (*bacA*), *bacillibactin* (*dhbA*), and *bacillaene* (*beaS*), biosynthesis genes in the genome of strain WS-10 based on strong antibacterial activity (34). PCR was carried out in a 25 μL reaction mixture containing 22 μL 1.1×-T3 super PCR mix (TSINGKE Co. Ltd. Beijing, China), 2 μL forward and reverse primers, and 1 μL genomic DNA. The PCR amplification conditions and primers used in this study for the amplification of lipopeptide and polyketide genes are described in Table S1.

### Pot experiment.

An open field pot experiment was conducted to investigate the biocontrol potential of strain WS-10 against tobacco bacterial wilt disease at Yongping County, Dali (25° 36′ N, 100° 16′ E), Yunnan Province, China, during the growing season May-September 2021. The average night/day temperature and rainfall during the growing periods were recorded as 15.2/24.5°C and 158 mm, respectively. Seedlings of flue-cured tobacco cultivar “Hongda” (highly susceptible) were transplanted in the pots (35 × 30 cm) filled with 10 Kg/pot of disease-free red soil. In addition, to overcome the nutrient deficiency, fertilizer was applied as base fertilizer (N-P_2_O_5_-K_2_O = 8-16-22) 45 g/pot at transplanting and after 20 days of transplantation (N-P_2_O_5_-K_2_O = 15-0-33) 25 g/pot as top fertilizer.

The experiment was conducted under three treatments: water as negative-control (CK), R. solanacearum WS-001 (T1), and combined R. solanacearum WS-001 + WS-10 (T2). Four weeks after transplantation at the 5 to 6 leaf stage, treatments T1 and T2 were inoculated with 50 mL/pot bacterial suspension of R. solanacearum WS-001 (1 × 10^7^ CFU/mL). Whereas strain WS-10 (1 × 10^7^ CFU/mL) was applied twice at 30 mL/pot in treatment T2 after 4 and 5 weeks of transplantation. R. solanacearum WS-001 and strain WS-10 were applied through the soil drenching method near the stem (24). According to the Tobacco Institute of Yunnan Standards, all integrated field management practices were maintained except disease management, as described by Tang et al. (4). The experiment was performed under a complete randomized design and repeated thrice with 15 plants per replication in each treatment.

### Assessment of disease incidence.

At the end of the experiment, disease incidence was recorded using a 0 to 9 disease rating scale as previously described by Hu et al. (43): plants with no visible symptoms (0), irregular necrotic spots on the stem, or few leaves wilted (1), black streaks developed on less than half of the stem height, or two-third of leaves wilted (3), black streaks developed on half of the stem height or more than two-third of leaves wilted (5), black streaks reaching on the top of the stem, or all leaves wilted (7), and death of whole plant (9). Following formulas were used to calculate disease incidence (Di), disease index (DI), and control effect (C.E): Di (%) = (*n*/*N*) × 100, DI (%) = (∑ [rating scale × *n*]/[*N* × highest rating scale]) × 100. C.E (%) = ([DI*_T1_*-DI*_T2_*]/DI*_T1_*) × 100. Here, *n* is the number of infected plants in the index, *N* is the total number of plants, DI*_T1_* is the disease index of treatment T1, and DI*_T2_* is the disease index of treatment T2.

### Colonization ability of Bacillus amyloliquefaciens WS-10 in the host plant.

**(i) Construction of *gfp*-labeled B. amyloliquefaciens WS-10.** A *gfp*-labeled B. amyloliquefaciens WS-10 strain (*gfp*-labeled WS-10) was constructed to investigate the colonization ability of strain WS-10 in the flue-cured tobacco plants and rhizosphere soil using the methodology of Ji et al. (63). The *gfp*-labeled WS-10 strain was then constructed by transferring the plasmid pGFP4412 into competent cells of B. amyloliquefaciens WS-10. For the transformation, a 10 μL DNA of plasmid pGFP4412 was added to 100 μL of B. amyloliquefaciens WS-10 competent cells in an ice-cold 2-mm electroporation cuvette (BTX, Taiwan). The plasmid pGFP4412 was electroporated into the cells using an Electroporation System (Bio-Rad, USA) set at 1.8 kV cm^−1^, 25 μF, 200 Ω, and 4.2 ms. The cells were then quickly shifted into LB broth medium, incubated at 30°C and 140 rpm for 4 h, and cultured on the LB medium plates containing kanamycin (50 μg/mL).

**(ii) Inoculation of flue-cured tobacco roots with *gfp*-labeled WS-10.** The g*fp*-labeled WS-10 strain was cultured in 50 mL of LB broth supplemented with kanamycin (50 μg/mL) for 24 h and inoculated into the roots of flue-cured tobacco cultivar Hongda seedlings. After 7 and 21 days, the colonization ability of g*fp*-labeled WS-10 was observed in the roots and after 21 days in the rhizosphere soil through serial dilutions. The root samples were cut into 0.5 to 1 cm lengths, placed on a slide, and visualized under a confocal laser scanning microscope (Leica Germany, Model no. dm2000).

### Soil samples collection, extraction of DNA, and assessment of Ralstonia solanacearum WS-001 population dynamics.

Rhizosphere soil samples were collected from each treatment (3 samples per treatment and each sample is a composite of rhizosphere soil of 5 plants/replication) at the end of the experiment for microbial diversity analysis, as described by Cai et al. (5). Bulk soil was removed by shaking the plants, and tiny soil particles adhering to roots were collected as rhizosphere soil samples using the brush (5) and stored at −80°C for further study. Total soil DNA was extracted using the PowerSoil DNA extraction kit (MO BIO Laboratories, Inc., Carlsbad, CA, USA) from 0.5 g of soil per sample (3 DNA per sample and combined to make one composite sample), and extracted DNA was stored at −80°C for future use. The population dynamics of R. solanacearum WS-001 in the rhizosphere soil of flue-cured tobacco under different treatments were assessed through qPCR amplification using R. solanacearum species-specific primer pair *Rsol_fliC* (Table S2) (1).

### Rhizosphere microbial diversity analyses.

The V3-V4 and ITS1 variable regions of 16S and ITS *rRNA* gene were amplified using primer pairs 341F/806R (Table S2) and ITS5-1737F/ITS2-2043R (Table S2) to analyze bacterial and fungal diversity, respectively (52). The PCR products were sequenced on an Illumina MiSeq platform at Tsingke Biotechnology Co., LTD. (Beijing, China). The raw sequenced reads obtained from the Illumina platform were quality controlled through Trimmomatic software (Version 0.33) and UCHIME (Version 8.1) at a 20% cutoff level (64, 65). The obtained clean reads were processed on a UPARSE pipeline to generate operational taxonomic units (OTUs) at a 3% dissimilarity level (66) and blasted against UNITE (https://unite.ut.ee/) (67) and SLIVA (http://www.arb-silva.de) (68) of fungi and bacteria, respectively, at a threshold level of 70% for species annotation.

### Bioinformatics analysis.

QIIME 2 was used to calculate the alpha diversity indices (Chao 1, Shannon, Simpson, and Shannon evenness) and beta diversity based on the Bray-Curtis distance matrix. The results of alpha and beta diversity for bacterial and fungal communities were visualized in boxplots using the R package “ggplot2” and principal coordinate analysis (PCoA) with a vegan package in R (v3.5.0), respectively. Permutational multivariate analysis of variance (PERMANOVA) was conducted using the adonis function from the vegan package in R (v3.5.0). The relative abundance (RA) rarefaction curves for OTUs, RA bar plots at phylum and genus level, chord diagram at the genus level, and heatmaps of beta-diversity based on Bray-Curtis distance matrix were generated using R scripts in R (v.3.5.0) (52). The Wilcoxon test was used in wilcox.test function in R to calculate the significant differences among microbial communities and was considered significant when *P* < 0.05. Co-occurrence network analysis was conducted using sparCC in R for OTUs at the genus level (*P* < 0.05 and correlation coefficient > 0.3) for bacterial and fungal communities. The network properties were calculated and visualized in Gephi 0.9.2. Correlation analysis was performed between bacterial-fungal genera and disease incidence according to Pearson correlation coefficient (PCC, *P* < 0.05) using the ggcor package in “ggplot2” and visualized through a heatmap. IBM SPSS Version 20.0 (SPSS Inc., Chicago, IL, USA) was used to calculate the significant differences among treatments according to Duncan's multiple range test at *P* < 0.05. All figures were adjusted, combined, and modified by Adobe Illustrator 2019.

### Data availability.

The data sets generated for this study can be found in NCBI public database. All sequences of ITS and 16S rRNA genes can be found in Sequence Read Archive (SRA) under BioProject no. PRJNA818326.
