# Supplementary material for: Plant-Microbe Interaction: Mining the Impact of Native Bacillus amyloliquefaciens WS-10 on Tobacco Bacterial Wilt Disease and Rhizosphere Microbial Communities
Source: Microbiol Spectr. 2022 Aug 1;10(4):e01471-22. doi: 10.1128/spectrum.01471-22 (PMC9430121; doi:10.1128/spectrum.01471-22)
Supplement: Supplemental file 1 — Table S1-S7 and Fig. S1-S5. Download spectrum.01471-22-s0001.pdf, PDF file, 0.8 MB [file spectrum.01471-22-s0001.pdf]

Plant-microbe interaction: Mining the impact of native *Bacillus amyloliquefaciens* WS-10 on tobacco bacterial wilt disease and rhizosphere microbial communities

Waqar Ahmed[1,2,3, #], Zhenlin Dai[2,3, #], Jinhao Zhang[2,3, #], Shichen Li[1,4], Ayesha Ahmed[2], Shahzad Munir[2], Qi Liu[2,3], Yujiao Tan[1,5], Guanghai Ji[2,3, *] and Zhengxiong Zhao[1, *]

[1]College of Resources and Environment, Yunnan Agricultural University, Kunming, 650201, Yunnan, China

[2]State Key Laboratory for Conservation and Utilization of Bio-resources in Yunnan, Yunnan Agricultural University, Kunming 650201, Yunnan, China

[3]Key Laboratory of Agro-Biodiversity and Pest Management of Ministry of Education, Yunnan Agricultural University, Kunming 650201, Yunnan, China

[4]College of Agronomy and Biotechnology, Yunnan Agricultural University, Kunming, 650201, Yunnan, China

[5]College of Tobacco Science, Yunnan Agricultural University, Kunming, 650201, Yunnan, China

[#]Authors contributed equally to this work.

***Correspondence authors**

Zhengxiong Zhao

E-mail: zhaozx0801@163.com

Guanghai Ji

E-mail: jghai001@163.com

Table S1; List of specific primers for the characterization of antimicrobial lipopeptides and polyketides biosynthesis genes.

Gene	Primer pair	Sequence (5'−3')	Annealing temperature (℃)
**Lipopeptides**			
ituC	ITUC-F	AGGATCCAAGCGTGCCTTTTACGGGAAA	59
	ITUC-R	AAAAAGCTTAATGACGCCAGCTTTCTCTT	
fenA	FenA-F	AAGAGATTCAGTAAGTGGCCCATCCAG	59
	FenA- R	CGCCCTTTGGGAAGAGGTGC	
srfA	SrfAA-F	AAAGGATCCAGCCGAAGGGTGTCATGGT	59
	SrfAA-F	AAAAAGCTTGTTTTTCTCAAAGAACCAGCG	
bmyA	BmyA-F	AAAGCGGCTCAAGAAGCGAAACCC	59
	BmyA- R	CGATTCAGCTCATCGACCAGGTAGGC	
**Polyketides**			
dfnA	DfnA-F	GGTGCGGCATGAAGATTTGAGATCACCG	59
	DfnA- R	GGAGAGCACTTCAATTCCGACGTTGACC	
bacA	BacA-F	GTGAAGGCCGTACTTTTGTCTGGC	59
	BacA-R	GGGGGGAAATACAGCTTCAGGGC	
dhbA	DhbA-F	CGCCTAAAGTAGCGCCGCCATCAACGC	59
	DhbA- R	CCGCGATGGAGCGGGATTATCCG	
beaS	BeaS-F	CGCAAAAGCTCTTCGACCGCCGTC	59
	BeaS- R	CTCTCGTGCCGTCGGAATATCCGC	

The PCR amplification conditions for lipopeptides and polyketides are as follow: initial denaturation at 94 ℃ for 7 min, followed by 40 cycles of denaturation at 94 ℃ for 1 min, annealing at 59 ℃ for 30 sec, and extension at 72 ℃ for 90 sec, and final extension at 72 ℃ for 12 min.

Table S2; Primers used in this study to determine *R. solanacearum* WS-001 population dynamic and soil microbial diversity analyses.

Gene	Primer pair	Sequence **(5'−3')**
	***R. solanacearum* WS-001 population dynamic**	
Rsol_fliC	*Rsol_fliC*- forward	GAACGCCAACGGTGCGAACT
	Rsol_fliC- reverse	GGCGGCCTTCAGGGAGGTC
	**V3-V4 region of bacteria**	
16S *rRNA*	341F	ACTCCTACGGGAGGCAGCAG
	806R	GGACTACHVGGGTWTCTAAT
	**ITS1 region of fungi**	
ITS *rRNA*	ITS5-1737F	GGAAGTAAAAGTCGTAACAAGG
	ITS2-2043R	GCTGCGTTCTTCATCGATGC

Table S3; Sequencing data processing for variable regions of bacteria (16S; V3-V4) and fungi (ITS1) rRNA.

Sample ID	Raw reads (#)	Clean reads (#)	Effective reads (#)	AvgLen (bp)	GC (%)	Q20 (%)	Q30 (%)	Effective reads (%)	OTUs
**Bacteria (16S; V3-V4)**									
CKR1	80097	80031	78586	417	56.66	99.60	98.14	98.11	972
CKR2	80040	79972	78599	416	57.21	99.62	98.19	98.20	913
CKR3	80032	79984	78241	415	56.56	99.62	98.21	97.76	997
T1R1	79827	79766	77429	418	56.05	99.61	98.14	97.00	1052
T1R2	80266	80174	77709	418	56.02	99.60	98.10	96.81	1104
T1R3	80025	79970	77639	418	56.27	99.60	98.14	97.02	1060
T2R1	79914	79848	77214	415	56.28	99.61	98.16	96.62	1091
T2R2	80024	79967	77250	415	55.94	99.63	98.21	96.53	1085
T2R3	79967	79894	78161	416	56.60	99.60	98.13	97.74	1058
Total	720192	719606	700828	Avg: 416					Avg; 1037
**Fungi (ITS1)**									
CKR1	67993	67922	67578	263	50.33	99.92	99.48	99.39	317
CKR2	80070	79990	79594	253	49.77	99.93	99.54	99.41	313
CKR3	67518	67451	67033	255	49.89	99.92	99.49	99.28	280
T1R1	79984	79919	78879	258	51.80	99.93	99.53	98.62	299
T1R2	52665	52619	52302	249	51.23	99.95	99.65	99.31	307
T1R3	115081	114663	111373	253	51.09	99.83	98.97	96.78	425
T2R1	42201	42164	41962	262	50.07	99.92	99.48	99.43	311
T2R2	79985	79917	79429	258	48.66	99.92	99.50	99.30	320
T2R3	75878	75818	75324	246	49.24	99.96	99.69	99.27	477
Total	661375	660463	653474	Avg: 255					Avg;339

Note: Raw reads stands for original reads. Clean reads are raw reads that filter low quality and short length sequences. Effective reads are the sequence of reads that are finally used for subsequent analysis after filtering chimeras. AvgLen is the average base pair length of effective reads. GC (%) represents the content of GC bases in effective reads. Q20 and Q30 are the percentages of bases with base quality values greater than 20 (sequencing error rate less than 1%) and 30 (sequencing error rate less than 0.1%) in effective reads. Effective reads (%) represents the percentage of the number of effective reads to the number of raw reads. **Here;** application of water (CK), application of *Ralstonia solanacearum* WS-001 (T1), and combined application of *R. solanacearum* WS-001, *Bacillus amyloliquefaciens* WS-10 (T2), and R1, R2, and R3 shows the number of samples per treatment.

Table S4; Alpha diversity indices of 16S rRNA and ITS gene for bacterial and fungal communities under different treatments at the at 97% similarity level.

**Sample ID**	**Chao 1**	**Simpson**	**Shannon**	**Shannon evenness**
		**Bacteria (16S; V3-V4)**		
CK	997.5±43.61^{b}	0.9792±0.08^{b}	7.56±0.14^{b}	0.7467±0.016^{b}
T1	1092.03±26.65^{ab}	0.9737±0.03^{b}	7.52±0.42^{b}	0.659±0.014^{c}
T2	1104.17±19.12^{a}	0.9861±0.02^{a}	7.91±0.10^{a}	0.8548±0.008^{a}
		**Fungi (ITS1)**		
CK	365.26±28.78^{c}	0.9403±0.12^{a}	5.53±0.29^{ab}	0.6541±0.011^{b}
T1	389.08±99.97^{b}	0.9167±0.46^{b}	4.99±0.85^{b}	0.5768±0.034^{c}
T2	413.35±80.23^{a}	0.9595±0.07^{a}	5.96±0.67^{a}	0.7370±0.012^{a}

Here; application of water (CK), application of *Ralstonia solanacearum* WS-001 (T1), and combined application of *R. solanacearum* WS-001, *Bacillus amyloliquefaciens* WS-10 (T2). Significant differences among treatments represents by different small letters within a column according to Wilcoxon-test at $p < 0.05$.

Table S5; Results of permutational multivariate analysis of variance (PERMANOVA) for bacterial and fungal communities based on Bray-Curtis distance matrix.

Pairs	*df*	Sum of Sqs	Mean Sqs	F. Model	R^2	*p*. value	Significance
**Bacteria (16S; V3-V4)**							
Group	2	0.28232	0.141162	3.0595	0.50491	0.007	**
CK-T1	1	0.163003	0.163003	3.687317	0.479662	0.1	ns
CK-T2	1	0.135934	0.135934	2.551482	0.389451	0.1	ns
T1-T2	1	0.12455	0.12455	3.04275	0.43204	0.1	ns
**Fungi (ITS1)**							
Group	2	0.52673	0.26337	2.043	0.40512	0.005	**
CK-T1	1	0.283631	0.283631	2.177238	0.352461	0.1	ns
CK-T2	1	0.313687	0.313687	2.569631	0.391138	0.1	ns
T1-T2	1	0.192777	0.192777	1.43452	0.263964	0.1	ns

Here; application of water (CK), application of *Ralstonia solanacearum* WS-001 (T1), and combined application of *R. solanacearum* WS-001, *Bacillus amyloliquefaciens* WS-10 (T2). Asterisks indicates significant differences at $*p < 0.05$, $**p < 0.01$, and $^{ns}p > 0.05$.

Table S6; Relative abundance of top 10 bacterial and fungal phyla in rhizosphere soil samples under different treatments (±SEM, n=3/treatment).

Phylum	CK	T1	T2
	**Bacterial (16S; V3-V4)**		
Proteobacteria	0.4124±0.0134[b]	0.5198±0.0036[a]	0.4379±0.0161[b]
Actinobacteria	0.2090±0.0013[a]	0.1600±0.0070[b]	0.1786±0.0105[b]
Acidobacteria	0.0977±0.0064[a]	0.0832±0.0012[b]	0.0856±0.0074[b]
Chloroflexi	0.0904±0.0094[a]	0.0675±0.0025[b]	0.0709±0.0129[b]
Firmicutes	0.0508±0.0089[a]	0.0333±0.0027[b]	0.0388±0.0070[b]
Patescibacteria	0.0372±0.0061[a]	0.0345±0.0058[a]	0.0407±0.0047[a]
Bacteroidetes	0.0272±0.0091[a]	0.0337±0.0033[a]	0.0354±0.0045[a]
Gemmatimonadetes	0.0109±0.0017[c]	0.0209±0.0021[b]	0.0282±0.0055[a]
Cyanobacteria	0.0118±0.0014[b]	0.0082±0.0001[b]	0.0381±0.0096[a]
Planctomycetes	0.0231±0.0029[a]	0.0153±0.0004[b]	0.0186±0.0009[b]
Others	0.0291±0.0031[a]	0.0234±0.0022[a]	0.0270±0.0030[a]
	**Fungal (ITS1)**		
Ascomycota	0.7143±0.0188[b]	0.8005±0.0044[a]	0.7323±0.0124b[b]
Basidiomycota	0.0619±0.0098[b]	0.0469±0.0075[c]	0.0800±0.0122[a]
Mortierellomycota	0.0171±0.0058[b]	0.0656±0.0058[a]	0.0618±0.0011[a]
Chytridiomycota	0.0550±0.0101[a]	0.0136±0.0008[b]	0.0490±0.0161[a]
Glomeromycota	0.0769±0.0205[a]	0.0104±0.0059[b]	0.0086±0.0005[b]
Rozellomycota	0.0013±0.0003[a]	0.0176±0.0120[a]	0.0058±0.0011[b]
Mucoromycota	0.0008±0.0003[a]	4.29183E-06±3.50426E-06[a]	0.00±0.00[a]
Olpidiomycota	0.00±0.00[a]	5.02879E-05±3.59319E-05[a]	0.0007±0.0006a
Zoopagomycota	4.04359E-05±3.30158E-05[a]	0.0006±0.0003[a]	7.71971E-05±3.86881E-05[a]
Neocallimastigomycota	4.83372E-05±2.80136E-05[a]	2.12736E-05±1.85233E-06[a]	0.0006±0.0004[a]
Others	0.0729±0.0145[a]	0.0446±0.0080[b]	0.0614±0.0156[a]

Here; application of water (CK), application of *Ralstonia solanacearum* WS-001 (T1), and combined application of *R. solanacearum* WS-001 and *Bacillus amyloliquefaciens* WS-10 (T2). Different small letters within a row indicates significant differences among treatments according to Wilcoxon-test at $p < 0.05$.

Table S7; Relative abundance of 10 most dominant bacterial and fungal genera in rhizosphere soil samples under different treatments (±SEM, n=3/treatment).

Genus	CK	T1	T2
**Bacterial (16S; V3-V4)**			
Chujaibacter	0.1070±0.0195[a]	0.1115±0.0110[a]	0.0614±0.0144[b]
Sphingomonas	0.0684±0.0158[b]	0.0461±0.0015[c]	0.0875±0.0148[a]
Ralstonia	0.0002±9.32455E-05[c]	0.0863±0.0200[a]	0.0097±0.0012[b]
Acidothermus	0.0332±0.0039[a]	0.0189±0.0010[b]	0.0175±0.0054[b]
Bacillus	0.0236±0.0027[a]	0.0152±0.0012[b]	0.0240±0.0051[a]
Bryobacter	0.0167±0.0017[a]	0.0156±0.0002[a]	0.0153±0.0013[a]
Phenylobacterium	0.0063±0.0010[b]	0.0147±0.0011[a]	0.0174±0.0020[a]
Granulicella	0.0080±0.0019[b]	0.0143±0.0019[a]	0.0156±0.0029[a]
Acidipila	0.0161±0.005[a]	0.0120±0.002[b]	0.0097±0.0039[b]
Pectobacterium	7.99393E-05±4.03084E-06[c]	0.0340±0.0058[a]	0.0036±0.0015[b]
Others	0.5906±0.0025[b]	0.5877±0.0067[b]	0.6310±0.0191[a]
Unclassified	0.0102±0.0014[b]	0.0073±0.0002[c]	0.0370±0.0096[a]
**Fungal (ITS1)**			
Penicillium	0.1728±0.0217[a]	0.1533±0.0390[a]	0.1009±0.0076[b]
Fusarium	0.1011±0.0238[b]	0.0675±0.0096[c]	0.1621±0.0250[a]
Humicola	0.0686±0.0108[ab]	0.0732±0.0138[a]	0.0631±0.0030[c]
Chaetomium	0.0470±0.0121[b]	0.1280±0.0513[a]	0.0254±0.0035[c]
Mortierella	0.0170±0.0057[b]	0.0655±0.0058[a]	0.0617±0.0012[a]
Arcopilus	0.0811±0.0297[a]	0.0076±0.0022[c]	0.0104±0.0039[b]
Trichoderma	0.0181±0.0042[c]	0.0529±0.0034[a]	0.0252±0.0062[b]
Saitozyma	0.0062±0.0025[c]	0.0141±0.0033[b]	0.0292±0.0039[a]
Condenascus	0.0208±0.0068[a]	0.0153±0.0103[a]	0.0036±0.0008[b]
Plectosphaerella	0.0006±0.0002[b]	0.0173±0.0062[a]	0.0174±0.0045[a]
Others	0.1770±0.0190[b]	0.1316±0.0409[c]	0.2120±0.0485[a]
Unclassified	0.2897±0.0253[a]	0.2736±0.0507[a]	0.2889±0.0420[a]

Here; application of water (CK), application of *Ralstonia solanacearum* WS-001 (T1), and combined application of *R. solanacearum* WS-001 and *Bacillus amyloliquefaciens* WS-10 (T2). Different small letters within a row indicates significant differences among treatments according to Wilcoxon-test at $p < 0.05$.

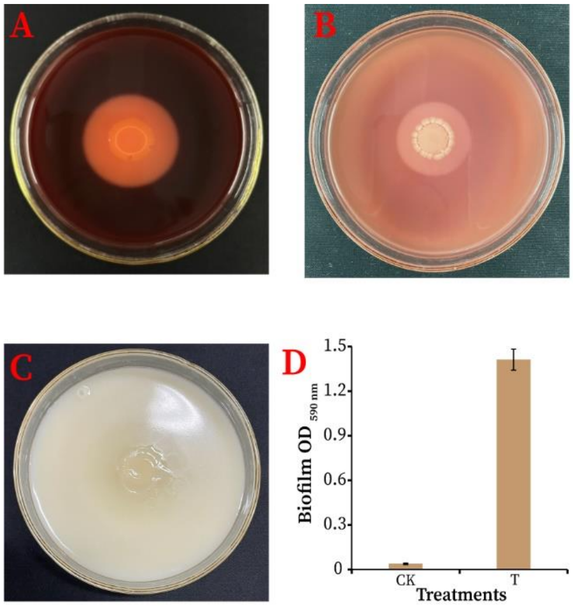

Figure S1; Activities of amylase, cellulase, protease synthesis and biofilm formation of *Bacillus amyloliquefaciens* WS-10. Here; amylase activity **(A)**, cellulase activity **(B)**, protease activity **(C)**, and biofilm formation **(D)**.

A

Properties	Activity
Amylase	+
Cellulase	+
Protease	–
EPS (mg·mL^{-1})	2.255±0.053
Biofilm	+

B

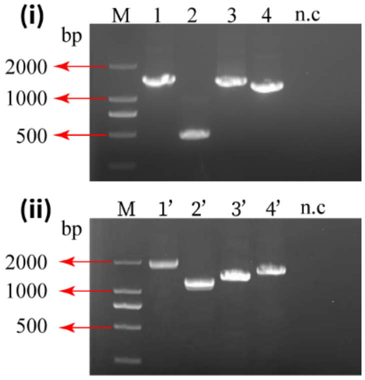

Figure S2; *In-vitro* assessment of biofilm formation, hydrolytic enzymes and exopolysaccharide production ability, and presence of antimicrobial biosynthesis genes. (A); Activities of amylase, cellulase, protease synthesis, exopolysaccharide (EPS) production, and biofilm formation. **(B);** Agarose gel electrophoresis images showing the presence of antimicrobial lipopeptides **(i)** and polyketides **(ii)** biosynthesis genes. **Here;** m = marker 2000 bp, 1-4: *fenA*, ituC, *srfA*, and *bmyA*, respectively **(i)**, 1’-4’: *dfnA*, *bacA*, *dhbA*, and *beaS*, respectively **(ii)**, and **n.c =** negative control.

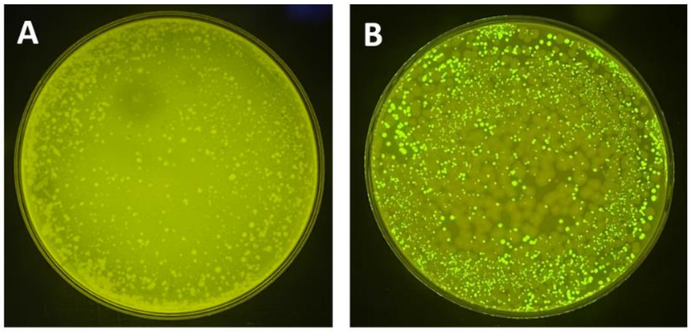

Figure S3; Colonization ability of *gfp*-tagged *Bacillus amyloliquefaciens* WS-10 in the rhizosphere of flue-cured tobacco plant. *B. amyloliquefaciens* WS-10 Without *gfp* as control **(A)** and *B. amyloliquefaciens* WS-10 tagged with *gfp* **(B)**.

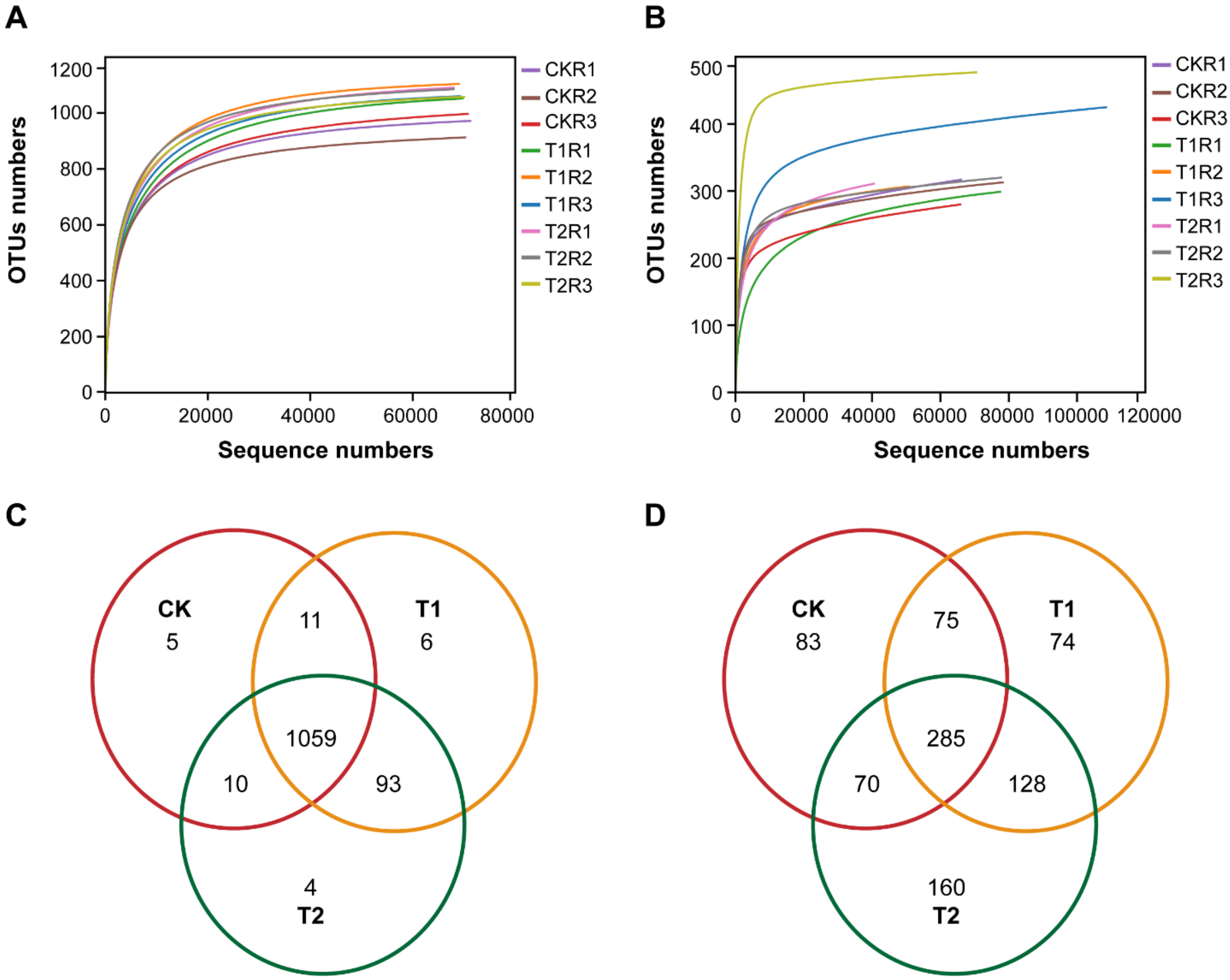

Figure S4; Rarefaction curves and operational taxonomy units (OTUs) analyses. Rarefaction curves generated from amplification of bacterial **(A)** and fungal **(B)** 16S and ITS1 genes of rRNA, respectively. Venn diagram shows the unique and shared OTUs of bacterial **(C)** and fungal **(D)** communities of rhizosphere soil under different treatments. **Here;** application of water (CK), application of *Ralstonia solanacearum* WS-001 (T1), and combined application of *R. solanacearum* WS-001 and *Bacillus amyloliquefaciens* WS-10 (T2).

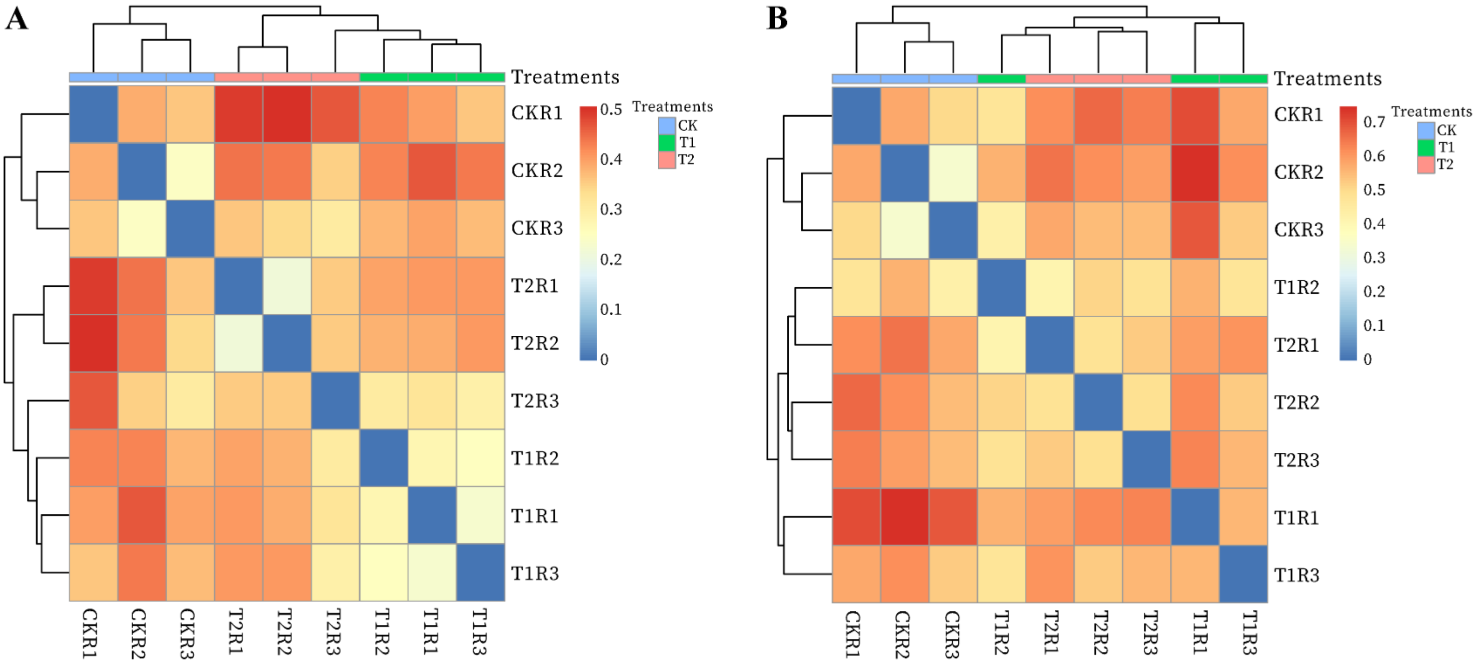

Figure S5; Bray-Curtis dissimilarity matrix heatmaps for bacterial (A) and fungal (B) communities structure under different treatments. Here; application of water (CK), application of *Ralstonia solanacearum* WS-001 (T1), and combined application of *R. solanacearum* WS-001 and *Bacillus amyloliquefaciens* WS-10 (T2).
